# Manipulation of Oxygen Tension in Damaged Regions via Hypoxia‐Induced IPN Hydrogel Microspheres for Intervertebral Disc Regeneration

**DOI:** 10.1002/advs.202417570

**Published:** 2025-04-15

**Authors:** Xingdie Zhou, Zhendong Lv, Zehao Chen, Yiming Xu, Chao Lin, Li Liu, Hao Chen, Bing Niu, Wenguo Cui, Yuhui Zhang

**Affiliations:** ^1^ Department of Spine Surgery Renji Hospital Shanghai Jiao Tong University School of Medicine 160 Pujian Road Shanghai 200127 P. R. China; ^2^ Department of Orthopaedics Shanghai Key Laboratory for Prevention and Treatment of Bone and Joint Diseases Shanghai Institute of Traumatology and Orthopaedics Ruijin Hospital Shanghai Jiao Tong University School of Medicine 197 Ruijin 2nd Road Shanghai 200025 P. R. China; ^3^ School of Materials Science and Engineering Shanghai University Nanchen Road 333 Shanghai 200444 P. R. China; ^4^ Department of Orthopaedics Laboratory of Key Technology and Materials in Minimally Invasive Spine Surgery Center for Spinal Minimally Invasive Research Hongqiao International Institute of Medicine Tongren Hospital Shanghai Jiao Tong University School of Medicine Shanghai 200336 China; ^5^ School of Life Sciences Shanghai University Nanchen Road 333 Shanghai 200444 P. R. China

**Keywords:** cell recruitment, hydrogel microspheres, microfluidics, oxygen tension

## Abstract

Disruption of low oxygen tension homeostasis during intervertebral disc degeneration inhibits endogenous stem cell viability and function, posing a challenge for endogenous regeneration. Here, to achieve sustained hypoxia manipulation, constructed hypoxia‐inducible interpenetrating polymer network (IPN) hydrogel microspheres (HIMS) are constructed by microfluidics to integrate the hypoxic system with a stabilizing network. The IPN is synthesized through a two‐step polymerization process, consisting of rapid photo‐crosslinked gelatin methacrylate anhydride (GM) polymer I and slow enzyme‐crosslinked vanillin‐grafted gelatin (GV) polymer II. The enzymatic reaction between GV and laccase is able to create a hypoxic microenvironment to modulate oxygen tension in situ within the injured region. HIMS can reduce microenvironmental oxygen tension by 1/3 and maintain a hypoxic microenvironment for up to 5 days, thereby activating the PI3K/AKT/HIF‐1α signaling pathway in endogenous stem cells to promote differentiation into nucleus pulposus‐like cells. Additionally, NSC‐Exos are loaded onto HIMS to trigger endogenous progenitor/stem cell recruitment and migration. Both in vitro and in vivo assays demonstrate that NSC‐Exos@HIMS facilitates stem cell recruitment, targets differentiation, and stimulates extracellular matrix synthesis. Overall, the microspheres established herein provide a novel strategy for manipulating oxygen tension and enhancing endogenous tissue regeneration in injured regions during intervertebral disc degeneration.

## Graphical abstract

A novel interpenetrating network hydrogel microsphere (NSC‐Exos@HIMS) is developed to manipulate oxygen tension in damaged regions and recruit endogenous stem cells. In vitro and in vivo results show that NSC‐Exos@HIMS maintains the hypoxia microenvironment for up to 5 days, which triggers nucleus pulposus cell‐like differentiation and enhanced ECM synthesis, providing a promising strategy for endogenous tissue repair.

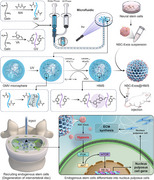

## 1 Introduction

Oxygen tension is a pivotal parameter essential for sustaining tissue health and functionality within biological systems, playing a critical role in maintaining tissue homeostasis. At the cellular level, oxygen tension is not only integral to the regulation of oxidative metabolism but also serves as an indispensable microenvironment for adenosine 5′‐triphosphate (ATP) synthesis and cellular viability.^[^ ^1^ ^]^ ATP, the cellular energy currency, relies on appropriate oxygen tension to ensure sufficient availability, thus facilitating cellular metabolic processes. Postnatal organisms have developed advanced hypoxia signaling pathways to precisely regulate and preserve this critical homeostasis.^[^ ^2^ ^]^ These signaling pathways detect alterations in oxygen tension in vivo and respond swiftly to ensure cellular physiological stability across diverse environments by promoting oxygen delivery and enhancing cellular hypoxia adaptation.^[^ ^3^ ^]^ Oxygen tension variations within the injured region may disrupt standard cellular metabolic pathways and impact cell fate determinations and overall tissue homeostasis. For instance, increased oxygen tension can lead to pulmonary and respiratory dysfunction, while a hypoxic microenvironment can foster disease progression in tumor metastasis^[^ ^4^ ^]^ and certain chronic diseases.^[^ ^5^, ^6^ ^]^ Although hypoxia‐inducing strategies have been employed to enhance stem cell differentiation and regenerative medicine, these methodologies often face significant limitations.^[^ ^7^, ^8^ ^]^ Many existing hypoxia induction methods struggle to achieve sustained regulation of oxygen tension, but the outcomes are often inconsistent. In addition, these methods may fail to replicate intricate physiological and pathological processes in vivo, thus compromising their clinical applicability. Therefore, precise modulation of oxygen tension within the injured region is imperative for preserving tissue homeostasis.

The intervertebral disc (IVD) is the body's largest avascular tissue, which obtains nutritional support by relying on oxygen and nutrients diffusing from the peripheral vasculature to the cartilaginous endplates.^[^ ^9^ ^]^ Intervertebral disc degeneration (IVDD) is a primary contributor to low back pain.^[^ ^10^ ^]^ Hypoxia, or reduced oxygen tension, is a defining characteristic of healthy intervertebral discs, which is critical for preserving the viability and functionality of nucleus pulposus (NP) cells. However, the inward growth of nerve fibers and blood vessels elevates oxygen tension during IVDD progression, thus disrupting oxygen tension equilibrium in the damaged region.^[^ ^11^, ^12^ ^]^ In a normoxia environment, the generation of reactive oxygen species (ROS) perturbs physiological homeostasis in the IVD, inducing NP apoptosis and advancing degeneration.^[^ ^13^ ^]^ Hypoxia‐inducible factor (HIF) is a pivotal molecule enabling cellular adaptation to microenvironmental oxygen tension and the regulation of cell fate.^[^ ^14^ ^]^ Variations in HIF, particularly HIF‐1α, directly affect the physiological condition of the IVD. Studies have shown that the HIF‐1α signaling pathway is downregulated in degenerated discs, indicating that heightened oxygen tension suppresses HIF‐1α expression in NP cells and contributes to disc degeneration. Additionally, diminished HIF‐1α levels can trigger ROS production, thus intensifying oxidative stress and potentially leading to apoptosis.^[^ ^15^, ^16^ ^]^ The recruitment and differentiation of endogenous stem/progenitor cells are critical mechanisms underlying spontaneous reparative processes post‐injury, with oxygen tension and HIF‐1α level directly influencing their functionality and fate. For instance, activating HIF‐1α can alleviate survival challenges of NP‐derived stem cells under compressive stress by enhancing autophagy.^[^ ^17^ ^]^ Hypoxic conditions have also been shown to make stem cells more inclined to differentiate into NP cells. Peroglio et al.^[^ ^18^ ^]^ demonstrated that thermoreversible hyaluronic acid‐based hydrogel significantly promoted the differentiation of human mesenchymal stem cells (MSCs) into NP‐like cells of the IVD under the hypoxic environment, as confirmed by in vitro and ex vivo organ culture experiments. Lee et al.^[^ ^19^ ^]^ demonstrated that supplying the hypoxic environment with neural crest cell‐derived conditioned medium significantly facilitated the differentiation of pluripotent stem cells to NP cells. Allon et al.^[^ ^20^ ^]^ co‐cultured MSCs and NP cell clusters under hypoxic conditions in a simulated IVDD environment, and observed a marked increase in glycosaminoglycan production, which further supports the role of hypoxia in tissue regeneration. Therefore, restoring oxygen tension homeostasis and HIF‐1α level is imperative for fostering endogenous regeneration of the IVD. However, the field of IVDD and regenerative engineering faces numerous challenges, including effective modulation of oxygen tension in the damaged region and efficient harnessing of endogenous stem cells for regenerative therapies.

Modulating microenvironmental oxygen tension homeostasis through bioactive materials represents a promising strategy for tissue regeneration. The existing methodologies for inducing cellular hypoxia include both physical and chemical approaches. Cobalt chloride (CoCl_2_) and deferoxamine (DFO) are well‐established chemical hypoxia inducers that stimulate HIF expression under normoxic conditions.^[^ ^21^, ^22^ ^]^ It was found in our previous study that HIF‐1α expression in alveolar endothelial cells was robustly activated by poly(lactic acid)‐hydroxyacetic acid copolymer (PLGA) nanoparticles encapsulating DFO, demonstrating potential therapeutic applications in bronchopulmonary dysplasia.^[^ ^23^ ^]^ Furthermore, we developed a pH‐responsive micro‐ and nano‐hydrogel system that achieved targeted bone tissue delivery by incorporating polyhedral oligosiloxanes (POSS) and conjugating DFO with the bone‐targeting peptide Asp8. This system can activate the HIF‐1α signaling pathway, mitigate bone resorption, and alleviate osteoporotic symptoms, thus offering a novel therapeutic strategy for preventing postmenopausal osteoporosis.^[^ ^24^ ^]^ However, chemical hypoxia agents elevate HIF‐1α/2α levels in a time‐ and dose‐dependent manner and exhibit cytotoxic effects at high concentrations, thus limiting their in vivo applications.^[^ ^25^ ^]^ Physical hypoxia offers superior biosafety, but directly reducing oxygen concentrations in the microenvironment via physical interventions remains a significant challenge. Park and Gerecht et al.^[^ ^26^ ^]^ developed a hypoxia‐inducible hydrogel using a Lac‐mediated oxygen depletion between catecholic acid and gelatin, achieving precise control of dissolved oxygen levels and gradients within 3D microenvironments. The crosslinking and oxygen depletion in current hypoxia‐induced hydrogels are limited by a single enzymatic reaction, creating a paradox between sustained hypoxia and the rate of gelation. The introduction of interpenetrating polymer network (IPN) injectable hydrogel presents a novel strategy in that it integrates rapid photo crosslinking with gradual enzymatic crosslinking methods. IPN not only extends the duration of the oxygen‐depleting enzymatic reaction but also maintains a stable hypoxic microenvironment through photo cross‐linking networks. Delivery of IPN hydrogel to the lesion site via minimally invasive injection promotes endogenous IVD regeneration and provides a new approach to treating degenerative diseases.

**Scheme 1:**
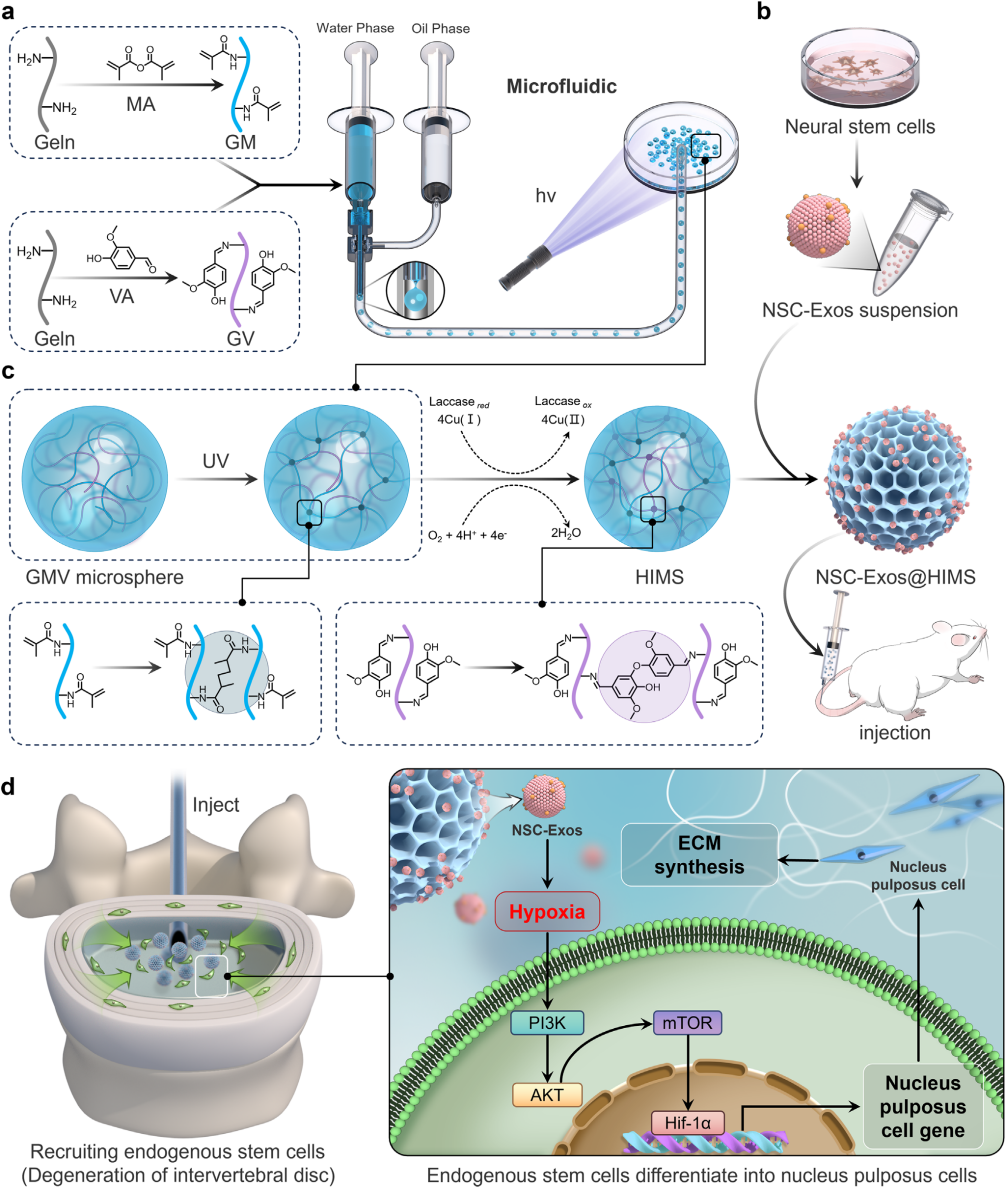
Schematic representation of the preparation of low oxygen tension‐inducing hydrogel microspheres (NSC‐Exos@HIMS). a) Microfluidic preparation of NSC‐Exos@HIMS. b) Harvesting exosomes from neural stem cells. c) The principle of double cross‐linking of interpenetrating network hydrogel microspheres and the minimally invasive drug delivery in intervertebral discs of SD rats. d) The mechanism of NSC‐Exos@HIMS via recruiting stem cells and manipulating low oxygen tension in the injured region to stabilize the microenvironment and promote nucleus pulposus regeneration.

In summary, to address the obstacle of impaired endogenous regeneration due to elevated oxygen tension in the degenerated disc microenvironment, we introduced a long‐term physically induced hypoxic IPN hydrogel microsphere system (NSC‐Exos@HIMS) (**Scheme** **1**). IPN was synthesized through a two‐step polymerization process. Initially, polymer I underwent photo crosslinking, followed by enzymatic crosslinking of polymer II in situ. This hydrogel microsphere combined rapid photo crosslinking with gradual enzyme‐mediated cross‐linking. Rapid gelation occurred in glycidyl methacrylate gelatin (GM) within seconds. Subsequently, vanillin‐grafted gelatin (GV) was produced via Schiff base reaction to initiate IPN synthesis by an enzymatic reaction between GV and laccase (Lac). This reaction created an in situ hypoxic environment that regulated oxygen tension in the damaged area. Additionally, NSC‐Exos were conjugated onto the hydrogel microspheres, serving as chemokines to recruit endogenous progenitor/stem cells in the IVD. This design integrated rapid photopolymerization with gradual enzymatic cross‐linking, giving NSC‐Exos@HIMS dual functionalities of both recruiting endogenous progenitor/stem cells and maintaining a sustained hypoxic microenvironment through Lac‐mediated oxygen‐depleting enzymatic reaction. This approach can promote stem cell differentiation into NP cells by activating the PI3K/AKT/HIF‐1α signaling pathway and stimulating extracellular matrix (ECM) synthesis, thereby fostering endogenous regeneration of IVDD.

## 2 Results and Discussion

### 2.1 Preparation and Characterization of HIMS

**Figure 1:**
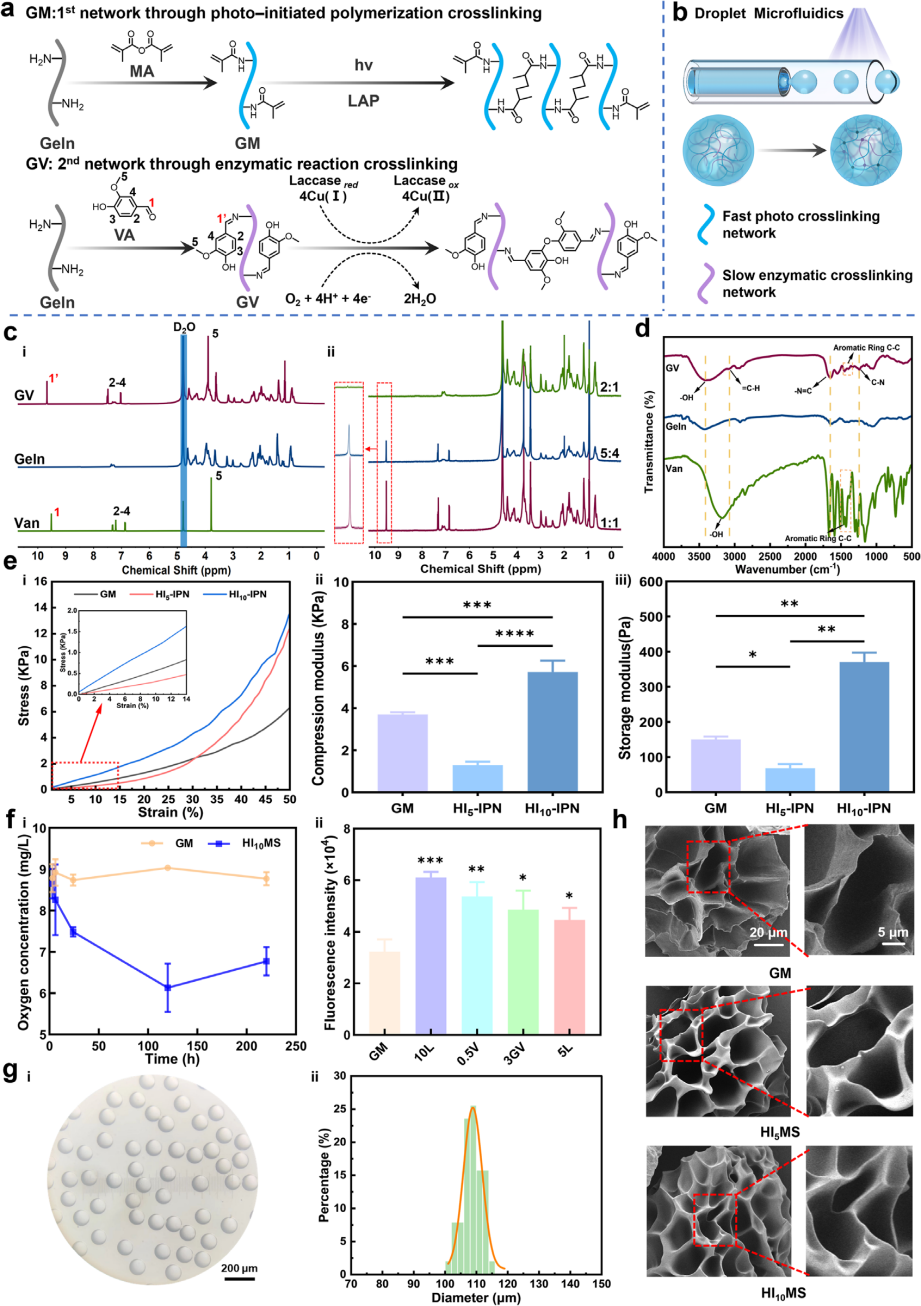
Characterization of interpenetrating network hydrogel microspheres with tunable oxygen tension in the damage region. a) Schematic diagram structure of photo crosslinking and enzyme crosslinking networks. b) Schematic diagram of microfluidics and structure of IPN microspheres. c) ^1^H NMR spectrum of the sample. i) GV, Geln, Van. ii) GV for Geln: Van cast ratio (w/w). d) FT‐IR spectra of GV, Geln and Van. e) Mechanical properties of hydrogel. i) Stress–strain curve of hydrogels. ii) Elastic modulus of hydrogels. iii) Rheological analysis of hydrogels. (*n* = 3). f) Characterization of oxygen consumption of microspheres. i) Long‐term oxygen consumption performance. ii) Effect of different ratios of microspheres on oxygen regulation. (*n* = 3). g) Morphological characteristics of microspheres. i) microscopic image of microspheres. ii) Size distribution of microspheres. h) SEM imagery of freeze‐dried microspheres. (*n* = 3). The data are presented as the mean ± SD. *p < 0.05, **p < 0.01, ****p < 0.001 and ****p < 0.0001.

In this study, we developed hypoxia‐inducible interpenetrating polymer network microspheres (HIMS) capable of modulating oxygen tension in damaged tissue regions (**Figure** **1**a). Based on prior work, photo cross‐linkable gelatin methacrylate (GM) was chosen as Polymer I for its highly tunable mechanical properties,^[^ ^27^ ^]^ knowing that gelatin‐formaldehyde derivatives can enhance the physicochemical properties of gelatin, including thermal stability and free radical scavenging activity in particular.^[^ ^28^ ^]^ Vanillin (Van), a natural phenolic compound, that exhibits antioxidant, antimicrobial, and anti‐inflammatory properties, was grafted onto gelatin via a Schiff base reaction.^[^ ^29^ ^]^ To replicate the hypoxic microenvironment, Lac‐mediated enzymatic reaction consuming O_2_ was used as polymer II. The IPN structure was prepared in two steps. First, GMV microspheres were prepared using a certain mechanical modulus by microfluidics (Figure 1b). Following UV cross‐linking, secondary enzymatic cross‐linking was conducted with variable cross‐linking degrees and oxygen modulation capacities.

For GV, ^1^H Nuclear Magnetic Resonance (^1^H NMR) spectra (Figure 1c‐i) revealed a new signal at *δ *= 9.67 ppm, representing the imine proton (─CH═N─) formed by the vanillin‐gelatin (GV) grafting, confirming successful Van grafting. The reaction was further validated by a change in the aldehyde proton's chemical shift. The Van substitution degree was tunable based on Van dosing ratios (Figure 1c‐ii). Meanwhile, Fourier transform infrared (FT‐IR) analysis was performed to confirm the successful grafting of Van onto gelatin (Figure 1d). The FT‐IR spectrum revealed characteristic absorption bands at 3418 cm^−1^ for gelatin attributable to the ─OH and ─NH_2_ groups in the gelatin's secondary amides (amide A).^[^ ^30^ ^]^ The GV spectrum displayed an absorption band at 3415 cm^−1^, indicative of the same interaction. Typically, Schiff bases produce ─C═N‐stretching vibrations at 1650–1600 cm^−1^, but this peak overlaps with the amide I band in gelatin (1644 cm^−1^).^[^ ^31^ ^]^ After crosslinking with vanillin, the intensity of the characteristic peak at 1644 cm^−1^ was enhanced. The aromatic ring C─C stretching vibrations were detected within the spectral range of 1400–1450 cm^−1^. In addition, the ═C─H stretching vibration in the aromatic ring was observed at 3080 cm^−1^ for GV. These observations from the FT‐IR analysis demonstrated the occurrence of chemical interactions between gelatin and Van.^[^ ^32^ ^] 1^H NMR and FT‐IR spectra demonstrated the successful preparation of GM (Figure S1‐i–iii, Supporting Information). The new signals at *δ *= 5.64 ppm and *δ* = 5.41 ppm, attributed to the vinyl proton with methacrylic anhydride, were observed in ^1^H NMR. The FT‐IR spectrogram of GM showed characteristic absorption peaks similar to those of gelatin.

Mechanical strength was verified through uniaxial compression and rheological tests. Detailed preparative formulations are shown in the supporting information (Table S1, Supporting Information). The results showed that the mechanical properties of HI‐IPN were lower than GM due to insufficient cross‐linking of the enzyme when the Lac concentration was 5 U mL^−1^, but they were significantly elevated when the Lac concentration was increased to 10 U mL^−1^ (Figure 1e‐i‐iii). Owing to its high cross‐linking density, GM demonstrated an enhanced mechanical strength. As the cross‐linking of hydrogel was directly dependent on the enzyme solution concentration, HI‐IPN hydrogels exhibited an improved mechanical strength at the enzyme concentration of 10 U mL^−1^ due to increased cross‐linking efficiency and a denser network structure enabled by Lac. This improvement may result from the increased generation of free radicals at higher Lac concentrations. These findings highlight the tunable mechanical properties of HI‐IPN hydrogels, indicating their capacity to maintain 3D structures and serve as a stable platform supporting cell growth and cellular functions.^[^ ^33^, ^34^, ^35^ ^]^ The complete rheological oscillation amplitude scanning curves are presented in the Supporting Information (Figure S2, Supporting Information). In addition, the visible light transmittance of HI‐IPN hydrogels at various Lac concentrations was compared with that of GM hydrogels (Figure S3, Supporting Information). We also measured the swelling curves of the hydrogels (Figure S4, Supporting Information). The swelling rate of the interpenetrating network hydrogel was much lower than that of the GM hydrogel. This is because as the number of cross‐linking points increases and the cross‐linking density increases, the 3D network of the gel becomes more compact and the lattice size of the cross‐linking network decreases, which in turn reduces the swelling capacity of the IPN hydrogel. In summary, we demonstrated that HI‐IPN hydrogels were successfully prepared with tunable oxygen consumption properties and a stable network structure.

Next, to precisely control microenvironmental oxygen tension, hydrogel microspheres were formulated per Table S2 (Supporting Information), with oxygen consumption properties of HI_10_MS and GM microspheres compared over time (Figure 1f‐i). It was found that the oxygen tension level in the GM group remained unchanged significantly, while HI_10_MS reduced the oxygen tension level significantly. The oxygen tension level of HI_10_MS was drastically reduced within the first 24 h and remained low for up to 5 days (the microenvironmental oxygen tension was reduced by a factor of 1.5), demonstrating that the oxygen‐depleting enzymatic reaction proceeded slowly. After day 5, the oxygen tension level increased gradually, indicating the completion of the enzymatic reaction. Subsequently, the oxygen concentration on day 5 was measured by fluorescence to explore the effect of changing the ratio of hydrogel components on oxygen regulation. An increase in fluorescence intensity indicated a decreasing oxygen content. It was found that the oxygen content decreased with the Van grafting rate, GV and Lac concentrations increasing (Figure 1f‐ii). This is caused by the Lac‐mediated O_2_‐consuming enzymatic reaction in which O_2_ was reduced to water when Lac reacted with the phenolic hydroxyl group (─OH) on Van, thus decreasing the oxygen content. This is a meaningful advantage because a prolonged low oxygen environment can be maintained based on the change of chemical parameters, making highly precise regulation of oxygen tension possible in damaged regions. Another strong advantage is that HI‐IPN hydrogels produce a hypoxic environment for a longer period (up to 120 h) compared to previously reported hypoxia‐inducible hydrogels (up to 1–12 h).^[^ ^26^, ^36^ ^]^ This facilitates HIF activation by HIMS to regulate the expression of a myriad of genes affecting life activities such as cell proliferation and differentiation.^[^ ^37^, ^38^, ^39^, ^40^ ^]^ Utilizing microfluidic technology, the microspheres demonstrated a superior size uniformity, featuring an average diameter of 100 µm, which is suitable for injection delivery (Figure 1g‐i,ii). Analysis of the scanning electron microscope (SEM) images for GM, HI_5_MS, and HI_10_MS, as depicted in Figure 1h, indicated that the microspheres possessed uniformly smooth exteriors and featured porous architectures. A decrease in pore size was associated with a higher degree of cross‐linking within the microspheres.^[^ ^41^ ^]^ Atomic force microscopy (AFM) and high‐resolution transmission electron microscopy (HRTEM) further validated the nanostructure and surface morphology of HIMS. With the increase of laccase concentration, the degree of cross‐linking was enhanced, the polymer network structure became denser, the molecular chain movement was restricted, and the microsphere surface tended to be homogenized.^[^ ^42^ ^]^ Therefore, the AFM results showed that the roughness of HIMS‐10 (Ra = 117 nm, Rq = 147 nm) was significantly lower than that of HIMS‐5 (Ra = 237 nm, Rq = 307 nm). In the TEM images, it can be seen with the corresponding results of AFM that HI_10_MS has a more homogeneous microstructure than HI_5_MS. (Figure S5, Supporting Information). These characterizations confirmed the successful preparation of HIMS.

### 2.2 Construction of Stem Cell‐Recruiting Microspheres

**Figure 2:**
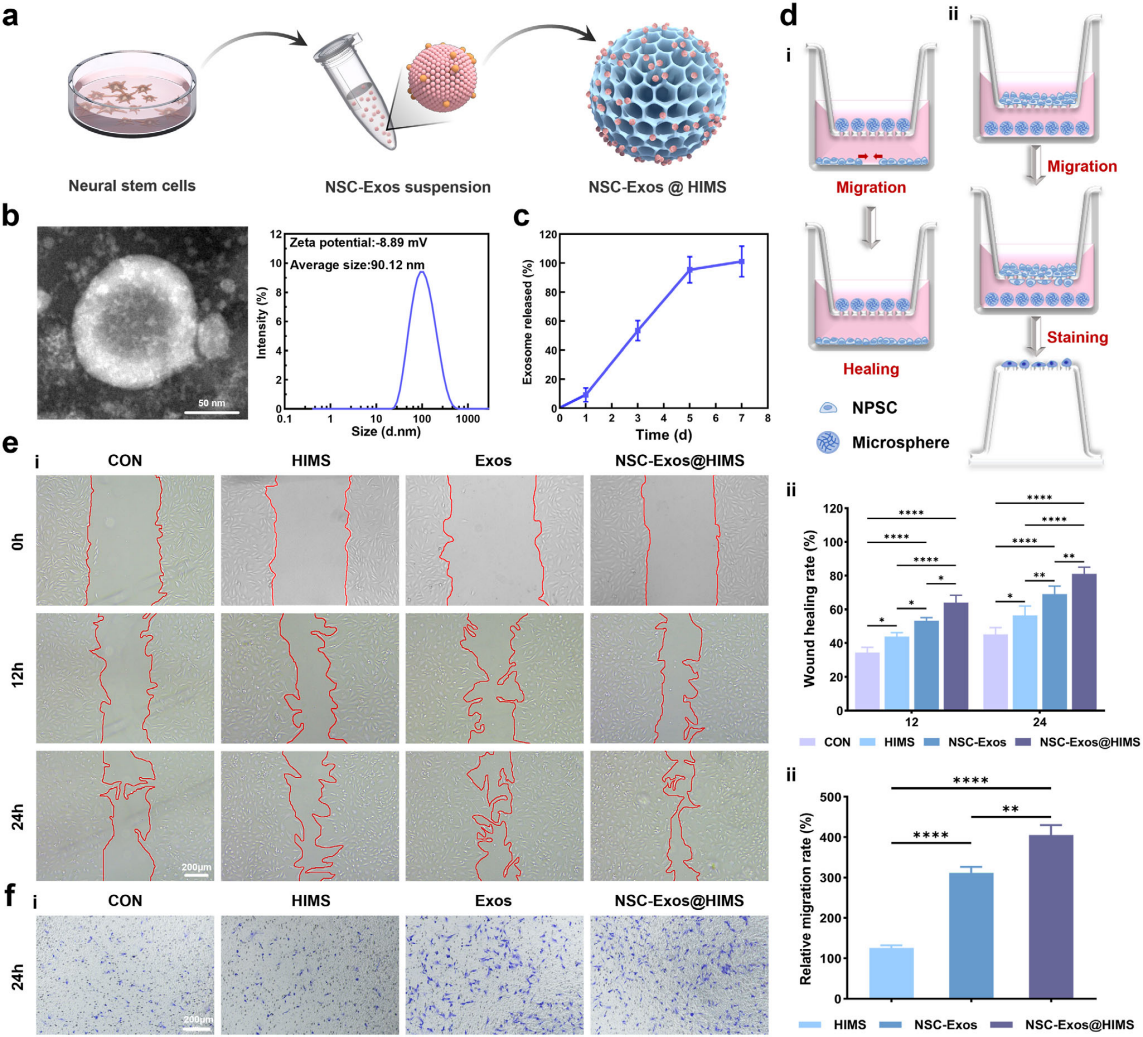
Assessment of cell migration‐promoting activity of microspheres. a) Extraction and loading of NSC‐Exos. b) TEM image, particle size distribution, and zeta potential of NSC‐Exos. c) Release curve of microsphere‐loaded NSC‐Exos. (*n* = 3). d) Schematic diagram of cell migration experiment. i) Wound healing assays. ii) Cell chemotaxis assays. e) Assessment of cell migration. i) microscopic images of scratch lines at 0, 12, and 24 h. ii) Quantification of wound healing percentages. (*n* = 3). f) Assessment of cell chemotaxis. i) microscopic images of the migration of NPSCs from the upper chamber at 24 h. ii) Quantification of migrating cell numbers. (*n* = 3). The data are presented as the mean ± SD. *p < 0.05, **p < 0.01, ****p < 0.001 and ****p < 0.0001.

IVD progenitor/stem cells have similar properties and functions to MSCs, including multipotency, self‐renewal capacity, and clonality. In addition, these cells exhibit greater tolerance to hypoxia and the acidic microenvironment than MSCs and therefore can serve as a promising candidate for IVD repair. Given the inherent disadvantages of stem cell transplantation, such as tumorigenicity, immunogenicity, and chromosomal aberrations, exosome delivery is a potential cell‐free method for recruiting endogenous stem cells.^[^ ^43^ ^]^ Research indicates that exosomes (Exos) can stimulate the paracrine signaling pathway, thereby attracting endogenous progenitor/stem cells to damaged regions and facilitating tissue regeneration.^[^ ^44^ ^]^ Therefore, to effectively recruit IVD progenitor/stem cells to the damaged region, we isolated and loaded NSC‐derived exosomes onto HIMS (**Figure** **2**a).

As shown in Figure 2b, the TEM images displayed cup‐shaped exosomes with diameters ranging from 30–200 nm. Dynamic light scattering analysis determined that NSC‐Exos had a mean diameter of 90.12 nm and a Zeta potential of −8.89 mV. NSC‐Exos were then bound to HIMS via hydrogen bonding. Additionally, micro/nano‐vesicles were bound and fused with HIMS due to high surface energy, which minimized the system's free energy, thus enabling the successful construction of NSC‐Exos@HIMS, designed for stem cell recruitment.^[^ ^45^ ^]^ The loading and distribution of NSC‐Exos was observed with rhodamine‐labeled NSC‐Exos (Figure S6, Supporting Information), exhibiting the high binding between NSC‐Exos and HIMS. A BCA protein assay was used to confirm the stable retention and sustained release of exosomes from HIMS. As shown in Figure 2c, the NSC‐Exos release rate was initially fast but slowed down after 5 day PBS incubation, with ≈90% of exosomes released from HIMS after 7 days. The protein calibration curve is shown in the Supporting Information (Figure S7, Supporting Information). The stable retention and slow‐release property make HIMS a promising platform for the efficient recruitment of progenitor/stem cells in IVD.

To assess the recruitment ability of microspheres, we utilized a Transwell co‐culture device to simulate cell migration within the stem cell niche.^[^ ^46^ ^]^ Wound healing and cell migration experiments demonstrated that the cell migration area expanded across all groups after 12‐ and 24‐h construction (Figure 2d‐i,ii;e‐i), especially in NSC‐Exos@HIMS group, where the wound healing rate reached 81.23% after 24 h (Figure 2f‐i). The Transwell migration experiment showed that NSC‐Exos@HIMS exhibited a significantly greater cell migration activity compared to HIMS (Figure 2e‐ii). The cell migration rate in NSC‐Exos and NSC‐Exos@HIMS groups was 3–4 times that in HIMS (Figure 2f‐ii), suggesting that neural stem cell‐derived exosomes are primary drivers for recruiting IVD progenitor/stem cells.

To further investigate the biocompatibility, microspheres were inoculated with nucleus pulposus stem cells (NPSCs) on anti‐adhesion culture plates and stained with Calcein AM/Propidium Iodide after 1, 3, and 5 day co‐culture. Despite minor cell death in some groups, most cells retained good morphology and viability, with survival >90% on microspheres (Figure S8‐i, ii, Supporting Information). In summary, hypoxia‐induced HIMS 3D culture of NPSCs maintains normal proliferative activity, which is a prerequisite for IVD regeneration engineering.

### 2.3 Evaluation of Stem Cell Differentiation Induced by HIMS

It was found that a hypoxic environment could stimulate the proliferation and differentiation potential of stem cells by activating HIF‐1α expression.^[^ ^47^, ^48^, ^49^ ^]^ Knowing that hypoxic (5% O_2_) conditions supported more glycosaminoglycan and collagen content in tissue‐engineered IVDs developed from human MSCs as compared to normoxic (21% O_2_) conditions,^[^ ^50^ ^]^ we next investigated the impact of HIMS‐induced oxygen tension reduction on NPSC differentiation. All microspheres were loaded with NSC‐Exos.

**Figure 3:**
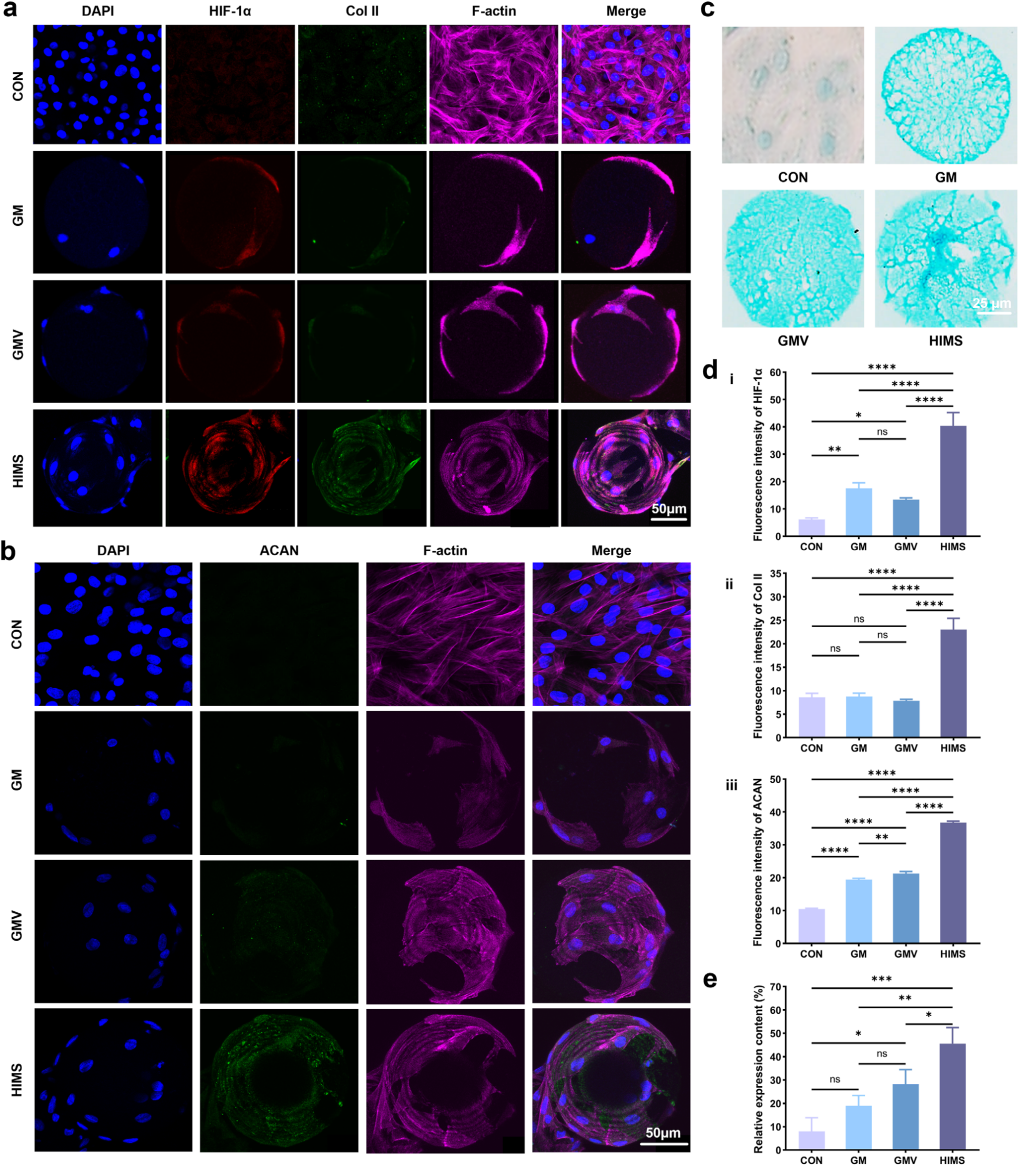
Impact of microspheres on nucleus pulposus‐like differentiation of NPSC. a) Immunofluorescence images of HIF‐1α and Col II. b) Immunofluorescence images of ACAN. c) Images stained with Alcian Blue. d) Quantification of the immunofluorescence intensity of HIF‐1α, Col II, and ACAN. (*n* = 3). e) Quantification of Alcian Blue staining. (*n* = 3). The data are presented as the mean ± SD. *p < 0.05, **p < 0.01, ****p < 0.001 and ****p < 0.0001.

We used immunofluorescence to evaluate the expression of HIF‐1α as a marker of cellular hypoxia responses (**Figure** **3**a). Due to the oxygen‐consuming process of the enzymatic crosslinking network of HIMS, we suggest that reduced oxygen tension could upregulate HIF‐1α expression in NPSCs. The results indicated that the expression of HIF‐1α was significantly activated in the HIMS group, and there was no statistical difference in the expression of HIF‐1α between GM and GMV groups (Figure 3d‐i), suggesting that the HIMS group could effectively activate hypoxic signaling in NPSCs.

To substantiate the role of hypoxic signaling in steering the differentiation of stem cells toward an NP phenotype, we evaluated the presence of markers associated with NP cells within NPSCs, as illustrated in Figure 3a,b. We focused on the expression of collagen II (Col II) and aggrecan (ACAN), which are known as major‐specific markers in NP cells.^[^ ^51^ ^]^ Quantification of Col II fluorescence intensity showed no statistically significant difference between changes in Control, GM, and GMV groups, but the HIMS group showed a statistically significant increase in Col II compared to all other groups (Figure 3d‐ii). In comparison to the other groups, a statistically significant increase in the expression of ACAN was observed in the HIMS group due to the addition of Lac (Figure 3d‐iii). This is closely related to the fact that oxygen‐depleting enzymatic reactions in HIMS greatly reduce oxygen tension in the microenvironment and activate hypoxic signaling. In addition, the results of the western blot further showed significantly higher protein expression of HIF‐1α in HIMS (Figure S9, Supporting Information). We further assessed proteoglycans (PG), which are known to be the major ECM components in NP, by Alcian blue staining. The GMV group does not affect microenvironmental oxygen tension levels, whereas the HIMS group undergoes an oxygen‐depleting enzyme reaction to reduce microenvironmental oxygen tension. The staining results (Figure 3c) showed that the staining in the HIMS group was deeper after 7 days induced differentiation and was significantly different from the control group. The GM and GMV groups were more lightly stained, without significant differences (Figure 3e). This suggested that the hypoxia microenvironment could induce the secretion of polysaccharide components in NP, in contrast to the oxygen‐rich microenvironment. The above results are consistent with the previous finding that the hypoxic condition could enhance the expression of the NP‐like cell markers and HIF‐1α in NPSCs.^[^ ^52^ ^]^ To summarize, hypoxic signaling activated by HIMS directs IVD stem cell differentiation toward NP cells in vitro and stimulates ECM synthesis.

### 2.4 Transcriptome Sequencing in NPSCs Treated with HIMS

**Figure 4:**
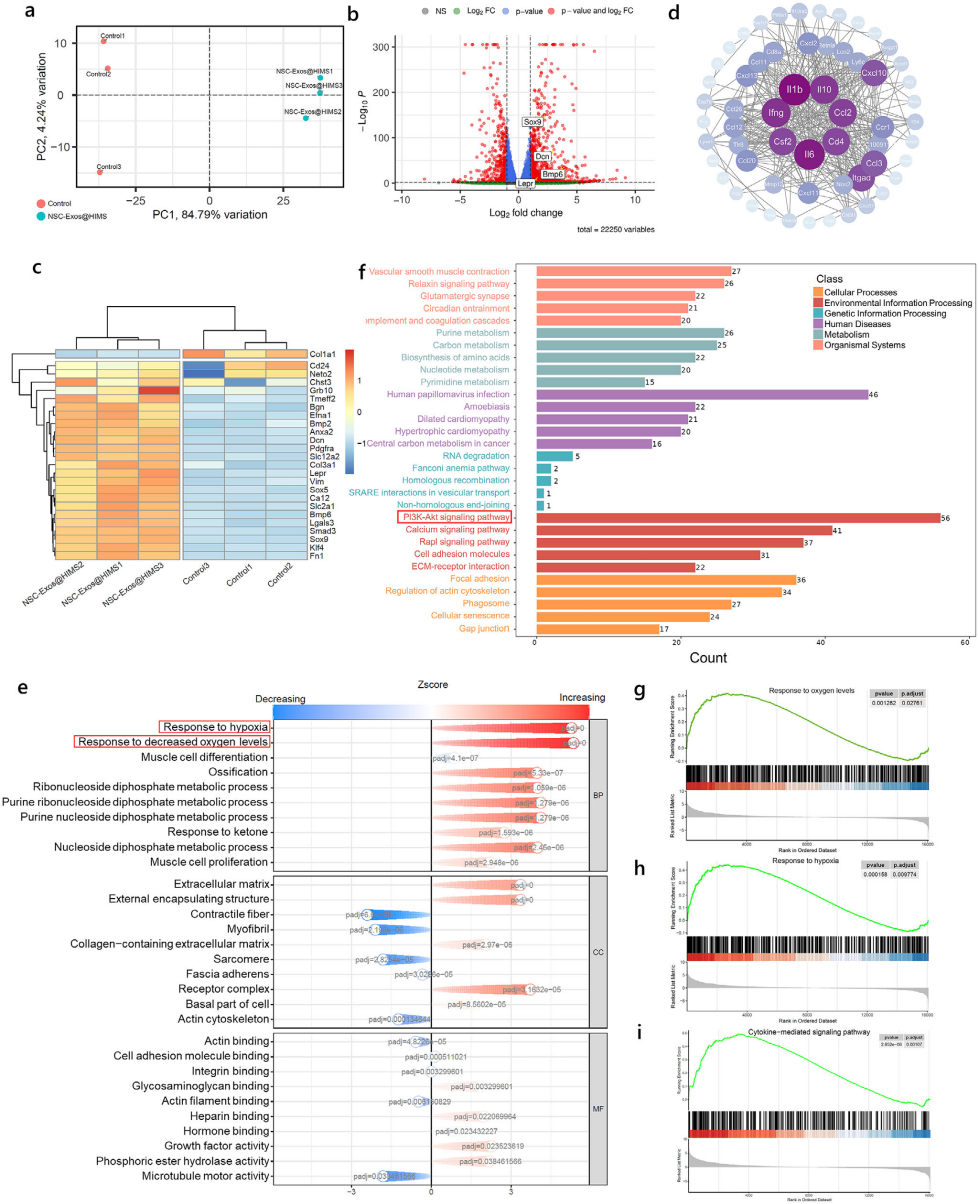
Transcriptome sequencing analysis. a) Principal component analysis. b) Volcano plots for transcriptome analysis of differentially expressed genes. c) Polymerization heatmap of stem cell differentiation‐related genes. d) Protein–protein interaction network analysis. e) GO enrichment analysis of differentially expressed genes. f) KEGG analysis of differentially expressed genes. g–i) Enrichment analysis of the KEGG‐related pathway GSEA set.

Transcriptome sequencing was performed to gain a better understanding about the underlying mechanism of NSC‐Exos@HIMS in directing NPSC differentiation and migration. Principal component analysis (PCA) was used to statistically analyze the samples from Control and NSC‐Exos@HIMS groups, and the results showed significant differences in gene expression between the samples (**Figure** **4**a). Comparison of differential expression between NSC‐Exos@HIMS and the Control group revealed a total of 1834 differentially expressed genes (DEGs), of which 1276 genes were up‐regulated and 558 genes were down‐regulated in the NSC‐Exos@HIMS group (Figure 4b). Then, we screened genes of interest by cluster analysis (Figure 4c) and found a significant number of DEGs between the Control and NSC‐Exos@HIMS groups. NSC‐Exos@HIMS greatly upregulated the expression of NP cell‐related genes such as Sox 9, NETO2, SLC2A1, and Anxa2.^[^ ^53^ ^]^ Besides, col1a1, which is known as an annulus fibrosus‐related gene, was downregulated in the NSC‐Exos@HIMS group. These results demonstrated that NSC‐Exos@HIMS could direct NPSC to differentiate into NP cells. The protein–protein interaction (PPI) network analysis was used to reveal the protein functions (Figure 4d). It was found that chemokines related‐protein network, including chemokine ligand 2 (CCL2), CCL3, CCL20, and CXC chemokine ligand 10 (CXCL10), were up‐enriched in NSC‐Exos@HIMS group (Figure 4d), demonstrating that NSC‐Exos enhanced stem cell migration by upregulating the expression of the chemokine family. CCL2 has been demonstrated as a hypoxia‐responsive target associated with cell migration and recruitment.^[^ ^54^ ^]^ Chemokine receptor 2 (CCR2) overexpressed MSCs exhibited enhanced directional migration and immunomodulation in response to CCL2.^[^ ^55^ ^]^ Therefore, we suppose that the combined effects of exosome release and hypoxia induction of NSC‐Exos@HIMS facilitate endogenous stem cell recruitment. Next, we analyzed DEGs via GO and KEGG enrichment to detect their functions and roles in NPSC differentiation. The GO functional enrichment result indicated that compared to the Control group, response to hypoxia, response to decreased oxygen level, extracellular matrix, and collagen‐containing extracellular matrix were up‐regulated (Figure 4e). KEGG enrichment analysis revealed that the PI3K‐AKT signaling pathway was up‐regulated in the NSC‐Exos@HIMS group (Figure 4f). The PI3K‐AKT pathway has been proved to be involved in chondrogenesis, and previous studies have reported that activation of the PI3K/AKT axis could enhance the chondrogenic differentiation of human MSCs.^[^ ^56^, ^57^ ^]^ Hence, we propose that NSC‐Exos@HIMS promoted NPSC targeted differentiation and ECM synthesis via the PI3K‐AKT signaling pathway. Gene set enrichment analysis (GSEA) also showed that the genes involved in the “response to hypoxia”, “response to decreased oxygen level” and “cytokine‐mediated signaling pathway” were significantly enriched in the NSC‐Exos@HIMS group (Figure 4g‐i). In addition, Western Blot results also showed that the protein levels of PI3K and AKT in NPSCs in the NSC‐Exos@HIMS group were significantly higher than those in the control group (Figure S10, Supporting Information), further demonstrating that NSC‐Exos@HIMS activated the PI3K/AKT axis. In summary, RNA sequencing results indicate that in the NSC‐Exos@HIMS group, the reduction of oxygen tension enhanced the migration and differentiation of NPSCs into NP cells by activating the response to hypoxia and the PI3K‐AKT signaling pathway.

### 2.5 HIMS Improves IVDD in SD Rats In Vivo

**Figure 5:**
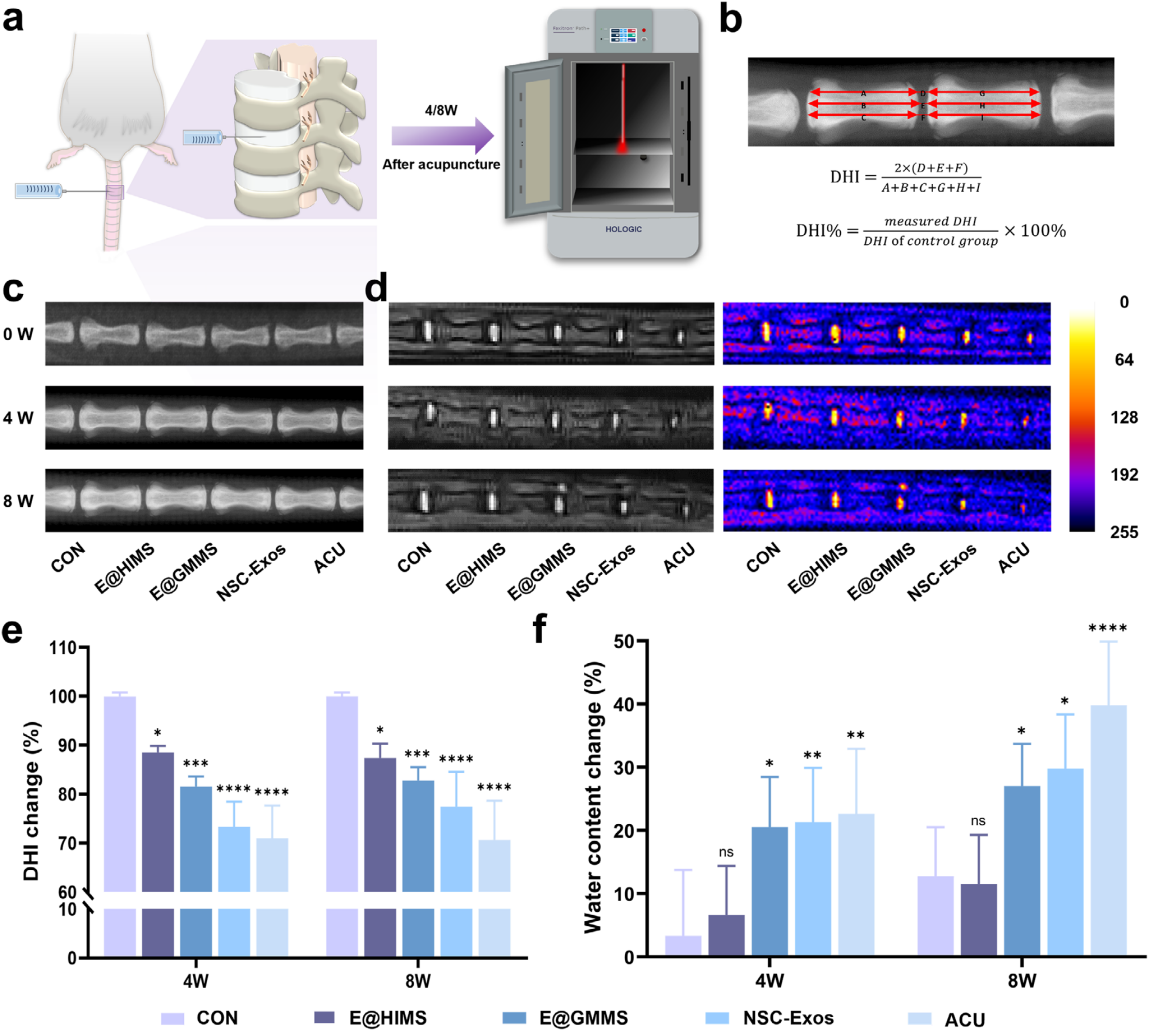
In vivo imaging assessment. a) Schematic diagram of the establishment of the rat caudal vertebrae puncture model and X‐ray imaging. b) Schematic diagram of intervertebral disc height calculation. c) X‐ray images of IVDs at 0, 4, and 8 W after surgery. d) MRI images and pseudo‐color images of the intervertebral disc at 4 and 8 W after surgery. e) DHI changes of the IVD at 4 and 8 weeks after surgery. (*n* = 6). f) Percentage decrease in disc water content at 4 and 8 weeks postoperatively compared to 0 weeks. (*n* = 6). The data are presented as the mean ± SD. *p < 0.05, **p < 0.01, ****p < 0.001 and ****p < 0.0001.

To investigate the effect of microspheres on treating IVDD in vivo, we built an IVDD rat model and observed IVDD progression and change in the disc height index (DHI) imageologically at 4 and 8 weeks post‐operation (**Figure** **5**a,b). To verify the effect of modulating low oxygen tension in the injured region on IVDD treatment, NSC‐Exos@HIMS (abbreviated as E@HIMS), NSC‐Exos@GMMS (abbreviated as E@GMMS), NSC‐Exos or PBS (Acupuncture group abbreviated as ACU) were injected into rat intervertebral discs for in vivo assessment after surgery. Changes in DHI intensity were observed via X‐ray imaging, and water content changes of the NP during the T_2_‐weighted phase were assessed by MRI (Figure 5c,d).^[^ ^58^ ^]^ At 4 weeks post‐surgery, rats in the ACU group exhibited significantly narrowed disc space and decreased T_2_ signal in the NP due to injury caused by needling. Compared to ACU, NSC‐Exos, and E@GMMS groups, the E@HIMS group exhibited an optimal treatment outcome, with a larger intervertebral space and a smaller reduction in water content which was not significantly different from the control group. At 8 weeks post‐surgery, the IVD space in rats of the E@HIMS group remained largely unchanged compared to those observed at 4 weeks, and the water content of the NP was reduced to a smaller degree, with no significant difference compared to the control group. In contrast, disc degeneration and intervertebral space atrophy continued deteriorating, and water content continued decreasing in rats of ACU, NSC‐Exos, and E@GMMS groups (Figure 5e,f). Although NSC‐Exos administration played a certain role in tissue regeneration initially, the continuous aggravation of inflammation and leakage of exosomes depleted its long‐term tissue regeneration potential. Although E@GMMS achieved a slow release of exosomes, it was unable to change the deteriorating oxygen tension microenvironment in the IVD, thus reducing the efficacy of IVDD treatment. In contrast, slow‐release exosome delivery in combination with fine regulation of oxygen tension produced a strong disc‐protective effect in the E@HIMS group, demonstrating that E@HIMS proposed in our study could further promote NP regeneration by modulating oxygen tension in the injured region and maintaining disc structural integrity and water content.

**Figure 6:**
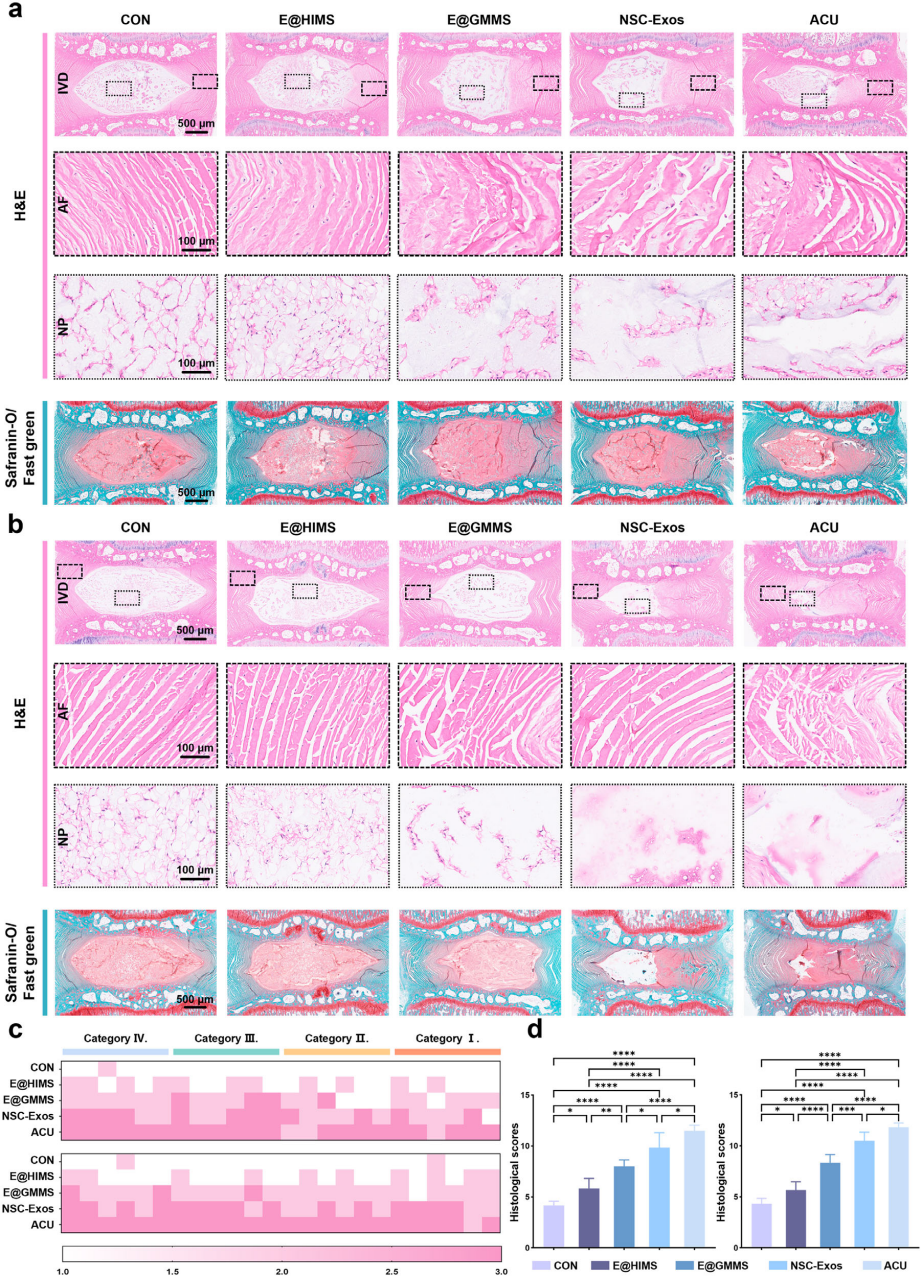
Histological analysis of animal experiments. a) H&E and safranin O/fast green staining images of IVDs at 4 W post‐operation. b) H&E and safranin O/fast green staining images of IVDs at 8 W post‐operation. c) Evaluation of histological scores based on four classifications at 4 (top) and 8 (bottom) W post‐operation. d) Cumulative histological scores at 4 (left) and 8 (right) W post‐operation (*n* = 6). The data are presented as the mean ± SD. *p < 0.05, **p < 0.01, ****p < 0.001 and ****p < 0.0001.

Next, we evaluated the IVD at 4 and 8 weeks post‐operation by histologic staining. Hematoxylin and eosin (H&E) staining was used to examine NP morphology and annulus fibrosus integrity. As shown in **Figure** **6**a,b, the retained area of NP was the largest in the E@HIMS group at 4 and 8 weeks post‐surgery, with the most intact disc structures and well‐defined NP and annulus fibrosus boundaries. However, the disc structures in the other treatment groups showed varying degrees of damage. It is worth mentioning that NP cells of the NSC‐Exos group appeared vacuolated or largely invisible by week 8, which is consistent with the imaging assessment. Safranin O/fast green staining was applied to primarily assess proteoglycan content and distribution, where the cartilage stains red and the bone stains green. As expected, the red staining intensity was decreased at 8 weeks post‐surgery as compared to that at 4 weeks. In addition, the proteoglycan expression in the E@HIMS group was always better than that in the other treatment groups. The IVD was then scored according to the criteria for histologic scoring of the IVD based on the previous methodology.^[^ ^59^ ^]^ The results at 4 and 8 weeks post‐operation showed the histologic score in the E@HIMS group was consistently lower than that in the other treatment groups, indicating a healthier disc status (Figure 6c,d). Although NSC‐Exos initially supported tissue regeneration, progressive inflammation, and exosome leakage depleted its potential over time. While E@GMMS achieved a slow release of exosomes, it did not mitigate the abnormal oxygen tension in the IVD microenvironment, thus limiting the therapeutic effect against IVDD. The E@HIMS group demonstrated the most effective disc regeneration by combining slow‐release exosomes with an improved oxygen tension microenvironment.

**Figure 7:**
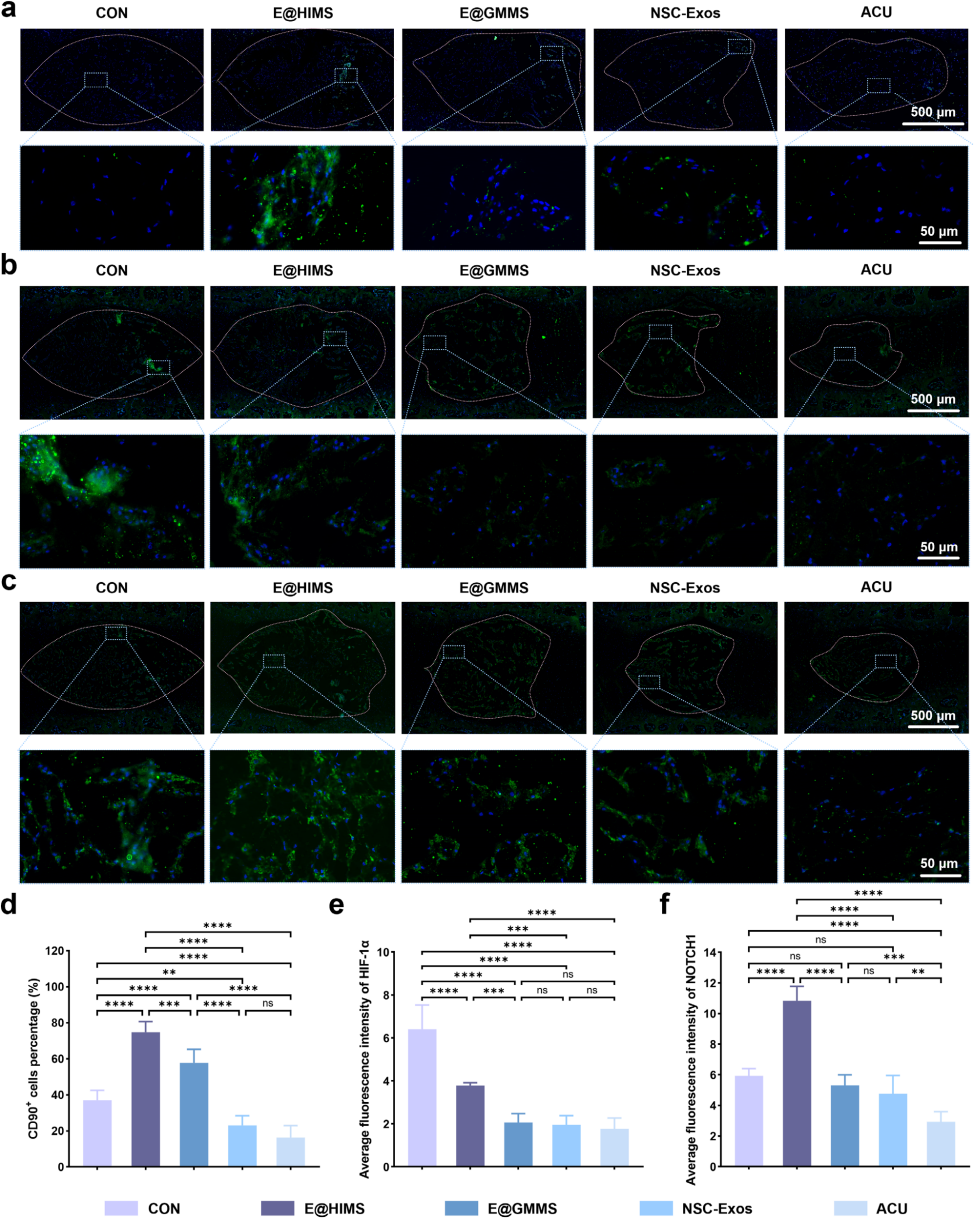
Immunofluorescence images of recruitment and differentiation of IVD endogenous stem cells. a) Immunofluorescence staining of CD90^+^. b) Quantitative analysis of the percentage of HIF‐1α. c) Immunofluorescence staining of NOTCH1. d) Quantitative analysis of the percentage of CD90^+^ cells (*n* = 6). e) Quantification of immunofluorescence intensity of HIF‐1α (*n* = 6). f) Quantification of immunofluorescence intensity of NOTCH1 (*n* = 6). The data are presented as the mean ± SD. *p < 0.05, **p < 0.01, ****p < 0.001, and ****p < 0.0001.

To further explore the therapeutic mechanism of the materials, immunostaining of CD90 (an IVD stem cell marker) was conducted on samples two weeks post‐surgery to trace progenitor/stem cells.^[^ ^60^ ^]^ Both E@HIMS and E@GMMS groups exhibited higher CD90^+^ cell percentages than the Control group, while the opposite trend was observed for free NSC‐Exos (Figure 7a,d). This may be attributed to the rapid diffusion of exosomes not encapsulated by the hydrogel scaffolds, thus limiting their ability to sustain effective concentrations at the injury site. Notably, the E@HIMS group had more significant CD90 cell enrichment than the E@GMMS group. The hypoxia‐inducible environment likely enhanced the migration capability of NPSCs and stimulate cell proliferation.^[^ ^61^, ^62^, ^63^ ^]^ Hypoxia was previously found to function as a regulator of stem cell and precursor cell differentiation.^[^ ^64^, ^65^ ^]^ Knowing that HIF‐1α maintains the stem cell phenotype and differentiation potential under hypoxic conditions by binding to NOTCH1 via activation of NOTCH signaling,^[^ ^66^, ^67^ ^]^ we explored the differentiation destiny of endogenous stem cells recruited by microspheres by immunofluorescence staining of HIF‐1α and NOTCH1(Figure 7b,c). The results showed that HIF‐1α expression in the E@HIMS group was the highest of all treatment groups (Figure 7e). We also observed that NOTCH1 fluorescence intensity followed a similar trend to CD90 cell percentages, with the highest expression in the hypoxia‐induced group (Figure 7f). Overall, these results suggest that hypoxia‐induced hydrogel microspheres create an optimized oxygen tension microenvironment that supports sustained exosome delivery and directed differentiation of recruited stem cells, thus significantly enhancing the disc regeneration potential.

## 3 Conclusion

To effectively manipulate oxygen tension in the damaged tissue, we developed a hydrogel microsphere known as NSC‐Exos@HIMS, with an interpenetrating polymer network designed to induce hypoxia through rapid photo crosslinking combined with gradual enzyme‐mediated crosslinking. This approach provides a novel strategy for modulating oxygen tension and promoting endogenous tissue regeneration through the synergistic effects of exosomes and physically induced hypoxia, while simultaneously recruiting and directing endogenous stem cell differentiation from the intervertebral discs. HIMS could sustain a hypoxic microenvironment for up to 5 days through the enzymatic activity of Lac. Our in vitro experiments demonstrated that these microspheres activated the PI3K/AKT/HIF‐1α signaling pathway in endogenous stem cells, thus promoting their differentiation into NP cells. Our in vivo study further confirmed that NSC‐Exos@HIMS upregulated the expression of NP‐related markers (e.g., COL II, Aggrecan) and HIF‐1α, and stimulated ECM synthesis, thereby promoting efficient endogenous tissue regeneration.

## 4 Experimental Section

### Synthesis of GM

The synthesis of methacrylated gelatin (GM) was carried out as detailed in prior report^s.[^ ^27^, ^68^ ^]^ Briefly, a 10% gelatin solution was prepared by dissolving type A pigskin gelatin (Sigma‐Aldrich) in carbonate buffer (pH 9.0) at 50 °C. After the gelatin was completely dissolved, methacrylic anhydride (Aladdin) was slowly added dropwise to the gelatin solution. After 3 h vigorous stirring, the reaction was terminated by adding excess phosphate buffer solution (PBS) to the system. The solution was dialyzed for 3 days in deionized water. After dialysis, the samples were freeze‐dried to obtain a white foam and then stored at −20 °C until needed for use. GM was characterized by ^1^H NMR (Bruker 600 M Hz, Swiss) and FT‐IR (Nicoleti S10, USA).

### Synthesis of GV

Gelatin‐vanillin (GV) with different vanillin (Van) contents was synthesized as previously described.^[^ ^28^ ^]^ Van was grafted onto the gelatin chain via a Schiff base reaction between the amino and aldehyde groups. Briefly, a 5 wt.% gelatin solution was prepared by dissolving gelatin in deionized water at 50 °C, during which anhydrous ethanol was added drop by drop to prevent polymer gelation. Meanwhile, different masses of Van were dissolved in anhydrous ethanol at room temperature and then added dropwise to the gelatin solution at 70 °C with stirring and reacted for 6 h. The reaction mixture was precipitated with acetone and washed with ethanol to remove unreacted monomer. The precipitate was then dissolved in deionized water and freeze‐dried. GV was characterized by ^1^H NMR (Bruker 600 M Hz, Swiss) and FT‐IR (Nicoleti S10, USA).

### Preparation of HIMS

Microspheres were prepared by a modified coaxial microfluidic device. The aqueous phase (GMV) was first prepared by dissolving lyophilized 5 wt.% GM, 5 wt.% GV, and 0.25 wt.% lithium triphenyl phosphate (LAP) in ultrapure water (37 °C), followed by its introduction into a dispersed phase channel. Paraffin oil, enriched with 5% weight Span 80 as an emulsifying agent, was fed into the continuous phase channel. A micro syringe pump (Longer Pump, LSPO1‐1A, UK) was utilized to regulate the flow rate ratio between the aqueous phase and the oil phase. GMV droplets were collected at −40 °C and crosslinked under 365 nm UV light for 5 min. Then microspheres were washed 3 times with ether and ultrapure water to remove paraffin oil and surfactant. GMV microspheres were sterilized with alcohol overnight. Lac (Aladdin) solution (5 and 10 U mL^−1^) was incubated with GMV microspheres overnight. Subsequently, the double cross‐linked HIMS was rinsed with PBS to eliminate any residual Lac.

### Physical Characterization of HIMS

Microspheres were observed under an optical microscope (Olympus, Japan) and their particle size distribution was counted. The freeze‐dried microspheres were characterized under SEM (Hitachi, Regulus8230, Japan), AFM (Bruker Dimension ICON, Germany), and HRTEM (JEOL JEM‐2100F, Japan) to observe the nanostructure and surface morphology. HI‐IPN hydrogels were analyzed in uniaxial compression (TA Q800, USA) to derive stress–strain curves. The compression modulus (*E*) of the hydrogel was determined by the slope of the initial linear section, and the compression modulus was determined based on the following equation:

(1) $$E=\frac{\Delta \varepsilon }{\Delta \sigma }$$

where *Δσ* is the amount of stress change and *Δε* is the amount of strain change. The rheological properties of the hydrogels were determined using a rheometer (Kinexus ultra+, UK). Specifically, the storage modulus (*G'*) was assessed over a strain range of 0 to 5% using an oscillatory amplitude test at a frequency of 5 rad s^−1^. The transmittance of different hydrogels was determined by a UV spectrophotometer (Jasco, V‐750, Japan). The hydrogel was placed in deionized water at 37 °C until a swelling equilibrium was reached. The rate of swelling was calculated according to the following equation:

(2) $$SR=\frac{W_{s}-W_{o}}{W_{o}}$$

where *W_s_* and *W_o_* are the weights of the swollen hydrogel and dried hydrogel.

### Measurement of the HIMS Ability in Regulating Oxygen Tension

HIMS and PBS solution (*v*/*v *= 1:2) were added to a 10 ml glass vial affixed with a patch sensor and sealed to prevent interference from outside air. Subsequently, the dissolved oxygen level in the PBS was monitored chronically with a fluorimetric oxygen analyzer (Presens, Fibox 4 trace, Germany) until the oxygen level no longer changed. Later, to facilitate the simultaneous examination of the effect of different components on the hydrogel's ability to regulate oxygen tension, the Oxygen Consumption Rate Fluorimetric Monitoring Kit (E‐BC‐F068) developed by Elabscience was chosen. Similarly, the hydrogel and PBS (*v*/*v* = 1:2) were added to a light‐avoiding 96‐well plate (Corning), and then the fluorescent probe was added and encapsulated with oil, and finally, the fluorescence intensity was measured with a fluorescent zymography instrument with excitation wavelength 405 nm and emission wavelength 675 nm (Varioskan LUX, USA).

### Isolation and Culture of Neural Stem Cells

As previously described, NSCs were isolated from the cerebral cortex of SD fetal rats at 14 days of gestational age under sterile conditions with minor modifications.^[^ ^69^ ^]^ Briefly, fetal rats were dissected to remove the cerebral cortex, and after fine stripping of the meninges, the brain tissue was minced with ophthalmic scissors and digested with 0.05% trypsin for 40 min at 37 °C. The digestion was then terminated by the addition of a trypsin inhibitor solution. The solution was filtered through a 40 µm cell strainer and centrifuged to remove excess digest. Cell pellet was resuspended in expansion medium containing DMEM/F12 (Gibco), 1% GlutaMAX (Gibco), 2% B27 supplement (Gibco), EGF (20 ng ml^−1^, PeproTech), FGF (20 ng ml^−1^, PeproTech) and 1% Penicillin‐Streptomycin (Gibco). Cells were counted and inoculated in 25 cm^2^ culture flasks and incubated at 37 °C in a 5% CO_2_ incubator.

### Extraction of Neural Stem Cell‐Derived Exosomes

Neural stem cells (NSCs) were cultured for 2 weeks to form neurospheres. The medium supernatant was collected and centrifuged at 500 × *g* for 5 min to remove cells. The supernatant was further centrifuged at 10 000–16 500 × *g* for 30 min and filtered through a 0.22 µm filter. Extracellular vesicle extraction reagent (Beyotime) was used to isolate NSCs‐derived exosomes according to the manufacturer's instructions.

### Characterization of Neural Stem Cell‐Derived Exosomes

A 10 µL drop of the exosome sample was placed on a carbon‐coated grid that was glow discharged in the air for 1 min, and then the grid was immediately negatively stained with 2% phosphotungstic acid for 60 s. The grids were examined using a transmission electron microscope (Hitachi, HT7800, Japan) at 80–120 kV. The size and distribution of exosomes were analyzed using a dynamic light scattering analyzer (Malvern, Nano‐ZS90, UK).

### NSC‐Exos Load and Release on HIMS

The exosome suspension was added and incubated with the sterilized microspheres overnight to prepare NSC‐Exos@HIMS. Subsequently, NSC‐Exos@HIMS was washed with PBS × 3 to remove unbound exosomes. NSC‐Exos@HIMS was soaked in PBS solution and placed in a constant temperature shaker at 37 °C. On days 1, 3, 5, 7, and 14, the exosome concentration in the PBS solution was determined using a BCA kit (Beyotime).

### Cell Biocompatibility

Biocompatibility assessment of HIMS microspheres. Sterilized microspheres were placed in 24‐well plates (Corning) and subsequently, NPSC was inoculated onto the microspheres at a density of 1 × 10^5^ cells well^−1^. On days 1, 3, and 5, the cellular viability and toxicity were assessed using the Calcein AM/PI Assay Kit (Beyotime), with an incubation period of 30 min, then immediately visualized and photographed under a laser confocal microscope.

### Fluorescence Staining of Cells for HIF‐1α, COLII and ACAN

Culture dishes were treated with a 0.75% polyvinyl alcohol (PVA) solution to create a barrier film, as detailed in prior reports, to inhibit cell attachment.^[^ ^70^ ^]^ The NPSC suspension was co‐cultured with microspheres loaded NSC‐Exos in treated culture plates. After 5 days incubation on microspheres, cells were washed with PBS × 3, fixed in 4% paraformaldehyde for 30 min, and permeabilized by adding 0.1% TritonX‐100 for 1 min. Cells were then incubated with primary antibodies to HIF‐1α, COL II, and ACAN (diluted 1:200 in the blocking solution) overnight at 4 °C. Fluor594 and FITC‐conjugated secondary antibodies (Affinity) were used to incubate with the cells for 60 min at room temperature. After three washes, cells were incubated with Fluor647‐conjugated Phalloidin (Gibco) and DAPI (Gibco) and observed using a laser confocal microscope (Olympus, FV3000, Japan). Fluorescence intensity was quantified using ImageJ.

### Western Blot Analysis of HIF‐1α

The cells were lysed in RIPA buffer, and protein concentrations were determined using a BCA assay. Proteins (20–30 µg) were separated by SDS‐PAGE and transferred to PVDF membranes. After blocking with 5% BSA, membranes were incubated with anti‐HIF‐1α (1:1000) antibodies overnight at 4 °C, followed by HRP‐conjugated secondary antibodies (1:5000) for 1 h. Proteins were detected using ECL, and band intensities were quantified with ImageJ. β‐actin served as a loading control.

### Alcian Blue Staining

NPSCs were cultured in anti‐adhesion well plates with GM, GMV, and HIMS microspheres. All microspheres were loaded with NSC‐Exos. After 7 days of differentiation, cells were fixed with 4% paraformaldehyde for 30 min at room temperature and then washed with PBS for 3 min. Samples were then paraffin‐embedded and cut into 5 µm sections. Alcian blue staining was performed using the Alcian blue staining kit (Cyagen, ALCB‐10001, China) under the guidance of the manufacturer's instructions.

### Cell Migration

A Transwell co‐culture device was established to assess cell migration. In the wound healing assays, sterilized HIMS, NSC‐Exos, and NSC‐Exos@HIMS were positioned in the upper compartment, and NPSCs were cultivated in the lower compartment (0.8 µm well, Corning). Once the cells achieved ≈90% confluence, a vertical prick was made in the center of the well using a 200 µL pipette tip. The complete medium (DMEM/F‐12 medium with 10% FBS) was replaced with a serum‐free medium. Wound healing was monitored at 0, 12, and 24 h using a bright‐field microscope, and the area of the scratch was measured using Image J. To assess migration capacity, sterilized HIMS, NSC‐Exos, and NSC‐Exos@HIMS were placed in the lower chamber, and NPSC was inoculated in the upper chamber (8 µm wells, Corning). The upper compartment was filled with serum‐free medium, while the lower compartment contained DMEM/F‐12 medium enriched with 10% FBS. After 24‐h incubation, cells on the chamber's membrane were fixed with 4% paraformaldehyde, stained with 0.5% crystal violet (Macklin), and examined under a bright field microscope (NIKON, Ts2R, Japan).

### RNA Extraction and Sequencing (RNA‐Seq) Analysis

NPSC were respectively cultured on flat dishes and NSC‐Exos@HIMS. Total RNA was extracted based on previous methods.^[^ ^71^ ^]^ The amount and purity of RNA for each sample were quantified using a NanoDrop 2000 spectrophotometer (Thermo Fischer Scientific, Wilmington, DE) and agarose gel electrophoresis. RNA sequencing was performed for each sample on an Illumina Novaseq 6000 (Wei Huan Biological Technology Co. Ltd, Shanghai, China) following the vendor‐recommended mRNA library construction and sequencing protocol. The gene expression was examined by calculating the FPKM (FPKM = [total _ exon _ fragments/mapped _ reads (millions) × exon _ length (kB)]) levels. DEGs were selected by R‐packet edgeR or DESeq2 with a fold change > twofold or a fold change < 0.5 and a *p*‐value < 0.05 and then analyzed for GO enrichment and KEGG enrichment.

### Western Blot Analysis of PI3K and AKT

The cells were lysed in RIPA buffer, and protein concentrations were determined using a BCA assay. Proteins (20–30 µg) were separated by SDS‐PAGE and transferred to PVDF membranes. After blocking with 5% BSA, membranes were incubated with anti‐PI3K (1:1000) or anti‐AKT (1:1000) antibodies overnight at 4 °C, followed by HRP‐conjugated secondary antibodies (1:5000) for 1 h. Proteins were detected using ECL, and band intensities were quantified with ImageJ. β‐actin served as a loading control.

### SD Rat IVDD Modeling

All animal procedures were approved by the Animal Ethics Committee of Shanghai University (Shanghai, China; Ethics Permission No. ECSHU 2024–077). SD rats aged 8 weeks were procured from Taizhou Yuxin Biotechnology Co., Ltd (Taizhou, China) and continued to be fed for 2 weeks. The rat tail IVDD model was established using the puncture method as described previously.^[^ ^27^ ^]^ SD rats were anesthetized by intraperitoneal injection using sodium pentobarbital (50 mg kg^−1^). After sterilizing the rat's tail, a 21G needle was used to puncture the centers of the 5–6, 6–7, 7–8, and 8–9 intervertebral discs, respectively. The needle was rotated 360° and then held for 30 s. Subsequently, 10 µL corresponding materials (NSC‐Exos@HIMS, NSC‐Exos @GMMS, NSC‐Exos, PBS) were injected locally using a 1 mL syringe.

### In Vivo Imaging Evaluation

At 0, 4, and 8 weeks post‐operation, X‐ray and MRI imaging of the rat caudal vertebrae were recorded. X‐ray images (Faxitron X‐ray, USA) were used to assess disc height, and the disc height was measured using ImageJ software. DHI% was calculated as previously described.^[^ ^72^ ^]^ The MRI scanner (MesoMR 23–060H‐I, China) was used to perform a T_2_‐weighted scan of the caudal region to assess the water content changes in the disc. Mean gray values were measured using ImageJ.

### Histologic Evaluation

After in vivo imaging evaluation, the disc samples were fixed in 4% paraformaldehyde for 24 h and decalcified with EDTA decalcification solution for 4 weeks. The decalcification solution was changed every two days. The samples were then paraffin‐embedded, cut into 5 µm sections, and stained with H&E and Safranin O and Fast Green. The tissue sections were finally examined under a bright‐field microscope (NIKON, Ts2R, Japan) and graded histologically.

### In Vivo Immunofluorescence Assessment

The expression of CD90, HIF‐1α, and NOTCH1 in the IVD was detected by immunofluorescence. The sections were permeabilized in 0.1% Triton X‐100 for 15 min, blocked in 5% goat serum for 30 min, and then incubated with CD90, HIF‐1α, and NOTCH1 primary antibodies (Affinity) (diluted 1:50 in the closure solution) at 4 °C overnight. Subsequently, the tissue sections were treated with the appropriate secondary antibodies linked to Fluor488 (Affinity), diluted at a ratio of 1:100 for 2 h at ambient temperature. The nuclear staining was performed using DAPI. Imaging was carried out by using a confocal laser scanning microscope (Olympus, FV3000, Japan).

### Statistical Analysis

All data were shown as the mean ± standard deviation (SD). The statistical analysis was performed using GraphPad Prism 8.0. The statistical differences were assessed by one‐way analysis of variance (ANOVA) and two‐way analysis of variance (ANOVA). *P *< 0.05 was considered statistically significant.

## Conflict of Interest

The authors declare no conflict of interest.

## Author Contributions

X.Z., Z.L., and Z.C. contributed equally to this work. X.Z. dealt with methodology, validation, formal analysis, investigation, and writing the original draft. Z.L. dealt with conceptualization, visualization, and writing the review and editing. Z.C. dealt with conceptualization, project administration, visualization, and writing the review and editing. Y.X., C.L., L.L., and H.C. dealt with the resources. B.N. dealt with funding acquisition. W.C. dealt with supervision, writing the review and editing, project administration, and funding acquisition. Y.Z. dealt with supervision, writing the review and editing, project administration, and funding acquisition.

## Supporting information



Supporting Information

## Data Availability

The data that support the findings of this study are available from the corresponding author upon reasonable request.
