# Supplementary material for: Manipulation of Oxygen Tension in Damaged Regions via Hypoxia‐Induced IPN Hydrogel Microspheres for Intervertebral Disc Regeneration
Source: Adv Sci (Weinh). 2025 Apr 15;12(22):2417570. doi: 10.1002/advs.202417570 (PMC12165120; doi:10.1002/advs.202417570)

**Supporting Information**

Manipulation of Oxygen Tension in Damaged Regions via Hypoxia-induced IPN Hydrogel Microspheres for Intervertebral Disc Regeneration

*Xingdie Zhou^†^, Zhendong Lv^†^, Zehao Chen^†^, Yiming Xu, Chao Lin, Li Liu, Hao Chen, Bing Niu*, Wenguo Cui*, and Yuhui Zhang*.*

X. Zhou, Z. Lv, H. Chen, Y. Zhang

Department of Spine Surgery, Renji Hospital, Shanghai Jiao Tong University School of Medicine, 160 Pujian Road, Shanghai 200127, P. R. China

E-mail: [zhangyuhui@renji.com](mailto:zhangyuhui@renji.com)

X. Zhou, Y. Xu, C. Lin, W. Cui

Department of Orthopaedics, Shanghai Key Laboratory for Prevention and Treatment of Bone and Joint Diseases, Shanghai Institute of Traumatology and Orthopaedics, Ruijin Hospital, Shanghai Jiao Tong University School of Medicine, 197 Ruijin 2nd Road, Shanghai 200025, P. R. China

E-mail: [wgcui@sjtu.edu.cn](mailto:wgcui@sjtu.edu.cn)

X. Zhou, L. Liu

School of Materials Science and Engineering, Shanghai University, Nanchen Road 333, Shanghai 200444, P. R. China

Z. Chen, C. Lin

Department of Orthopaedics, Laboratory of Key Technology and Materials in Minimally Invasive Spine Surgery, Center for Spinal Minimally Invasive Research, Hongqiao International Institute of Medicine, Tongren Hospital, Shanghai Jiao Tong University School of Medicine, Shanghai 200336, China

B. Niu

School of Life Sciences, Shanghai University, Nanchen Road 333, Shanghai, 200444, P. R. China

E-mail: [bingniu@shu.edu.cn](mailto:bingniu@shu.edu.cn)

† These authors contributed equally to this work.

**Supporting Tables**

**Table S1.** Scheme of microgels with different elastic modulus.

| Group | The concentration of precursor solution | | | | UV exposure time (min) |
| --- | --- | --- | --- | --- | --- |
|  | GM | LAP | GV | Lac (U·mL^-1^) |  |
| GM | 5% | 0.25% | 0 | 0 | 5 |
| HI_5_-IPN | 5% | 0.25% | 5% | 5 | 5 |
| HI_10_-IPN | 5% | 0.25% | 5% | 10 | 5 |

**Table S2.** Scheme of microgels with different oxygen control effects.

| Group | The concentration of precursor solution | | | | | UV exposure time (min) |
| --- | --- | --- | --- | --- | --- | --- |
|  | GM | LAP | GV | GV for Geln: Van (w/w) | Lac (U·mL^-1^) |  |
| GM | 5% | 0.25% | 0 | \ | 0 | 5 |
| HI_10_MS (10L) | 5% | 0.25% | 5% | 1:1 | **10** | 5 |
| 0.5V | 5% | 0.25% | 5% | **1:0.5** | 10 | 5 |
| 3GV | 5% | 0.25% | **3%** | 1:1 | 10 | 5 |
| HI_5_MS (5L) | 5% | 0.25% | 5% | 1:1 | **5** | 5 |

**Supporting Figures**

**Figure S1. The synthesis reaction and characterization of GM.** i)**.** The synthesis reaction of GM. ii) ^1^H NMR spectrum of Geln and GM. iii) FT-IR spectra of Geln and GM.

**
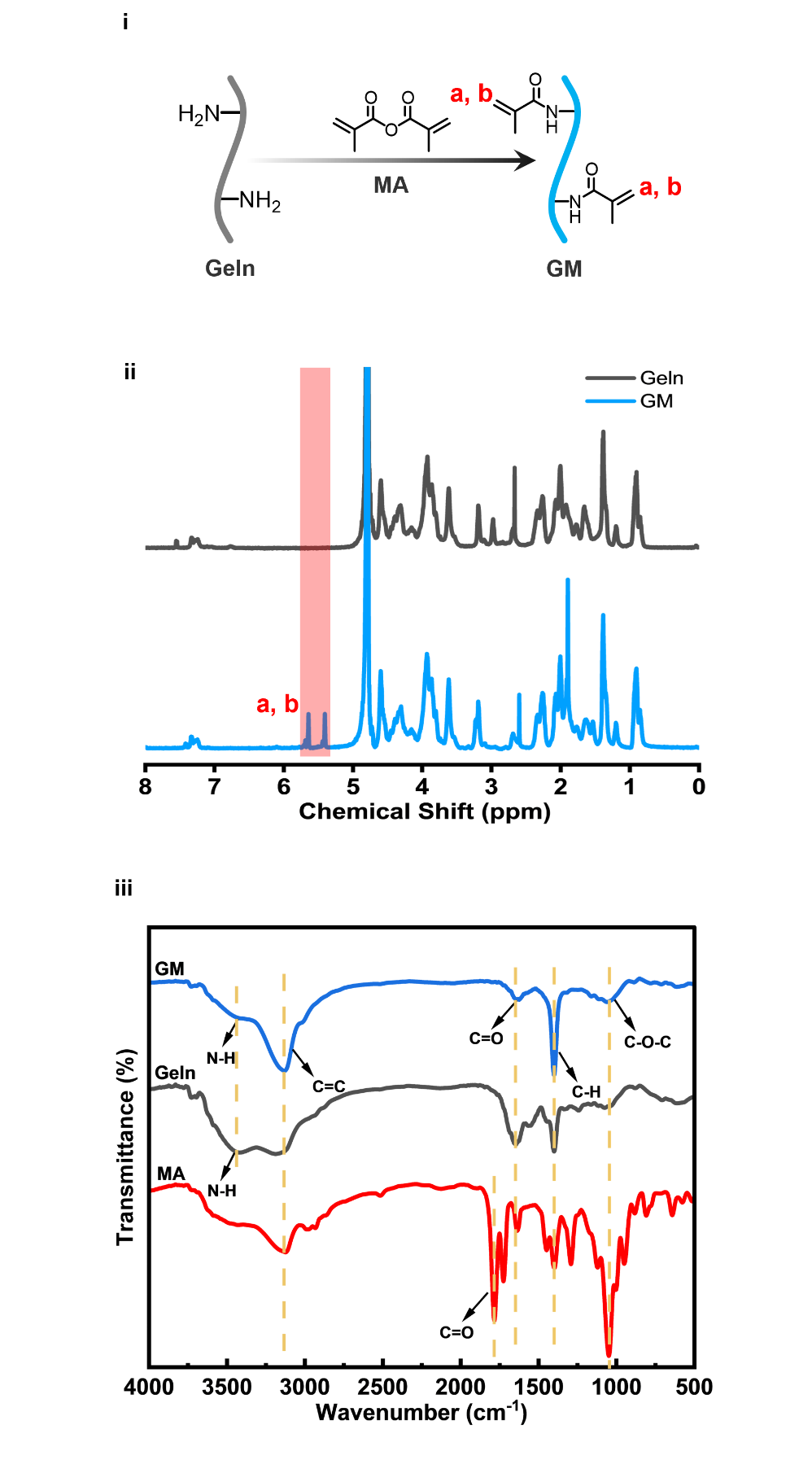
**

**Figure S2. The oscillatory amplitude sweeps curve of the hydrogels.**


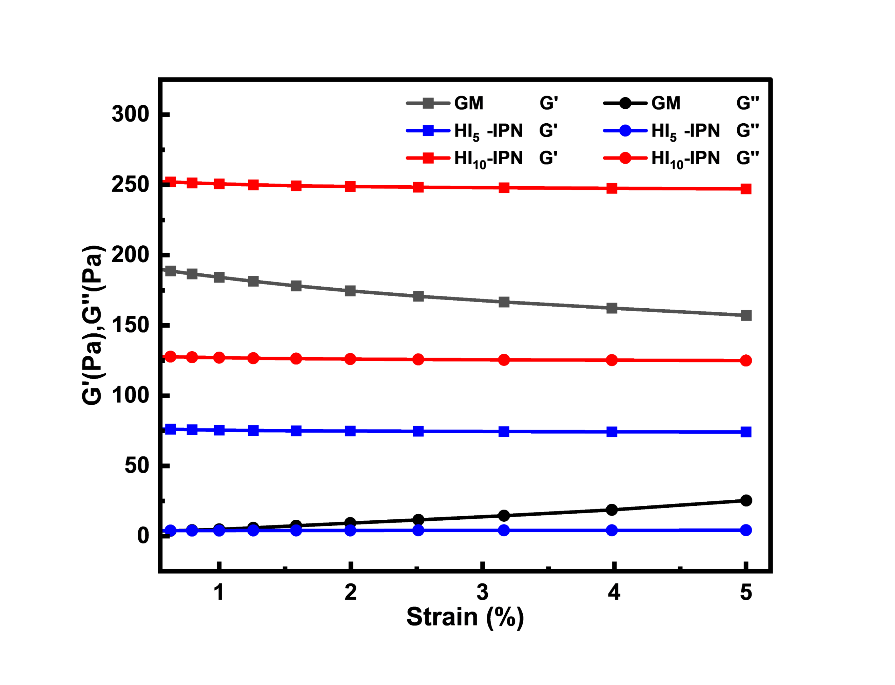


**Figure S3. Hydrogel transmission rate of different laccase concentrations.** i) Hydrogel object picture. ii) Transmittance of hydrogel in the visible range (400-800 nm). iii) Transmittance of 365 nm and 550 nm. (*n* = 3) The data are presented as the mean ± SD. ****p < 0.001 and ****p < 0.0001.


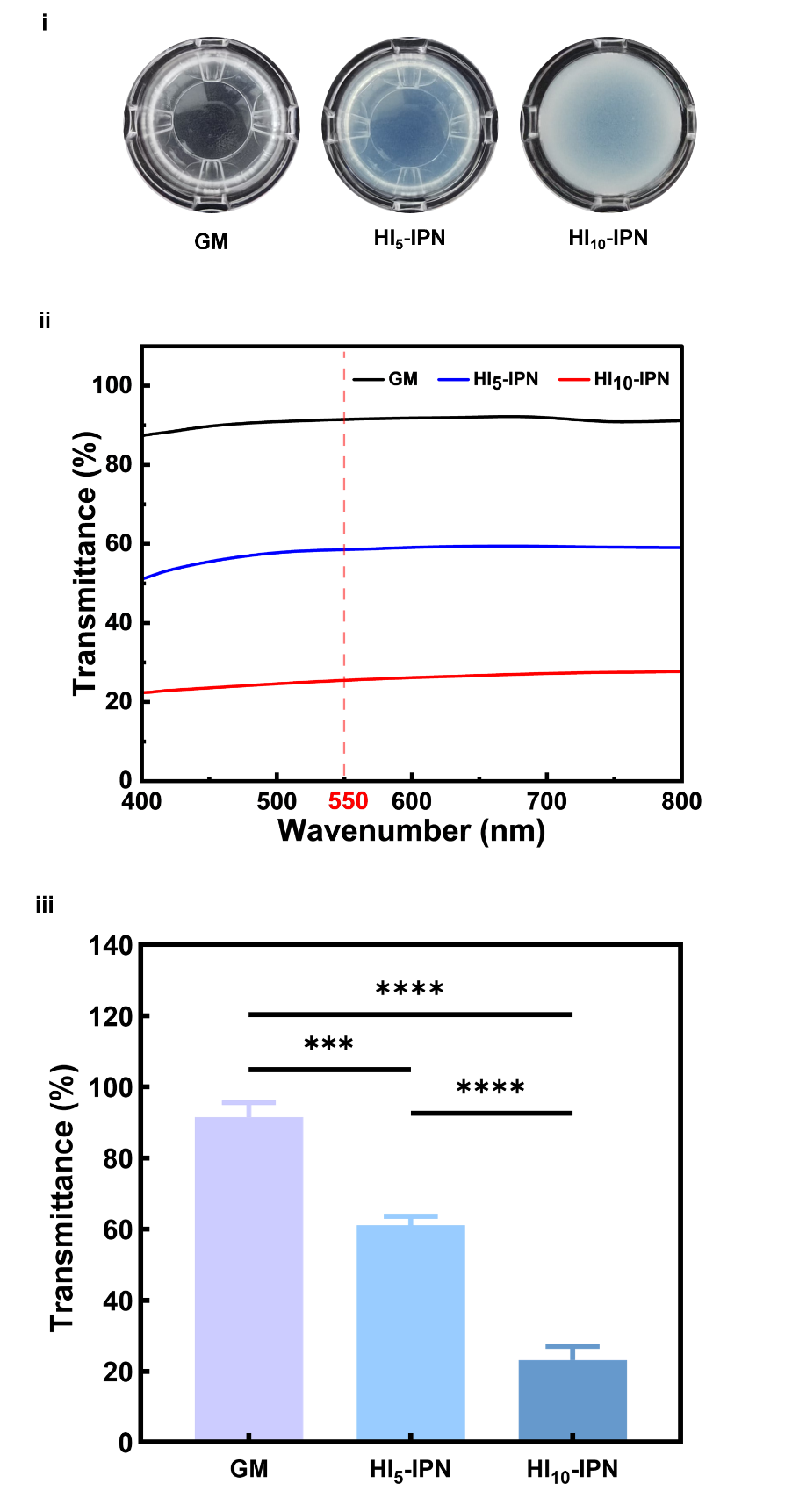


**Figure S4. IPN hydrogel swelling curve.**

**
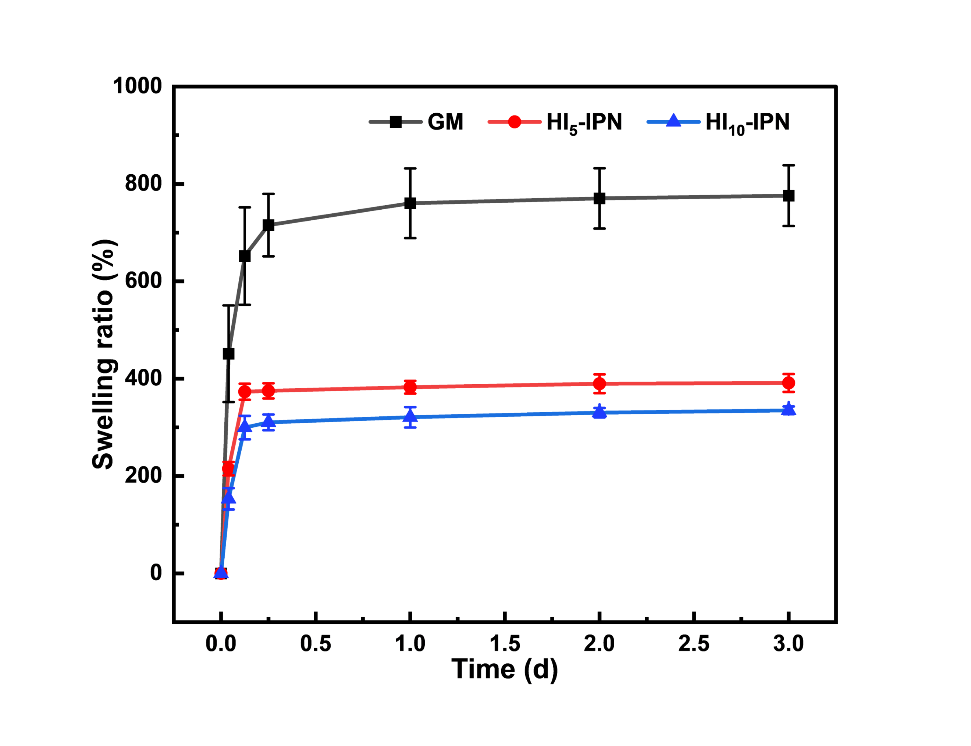
**

**Figure S5. AFM (i) and HRTEM (ii) images of HIMS.**

**
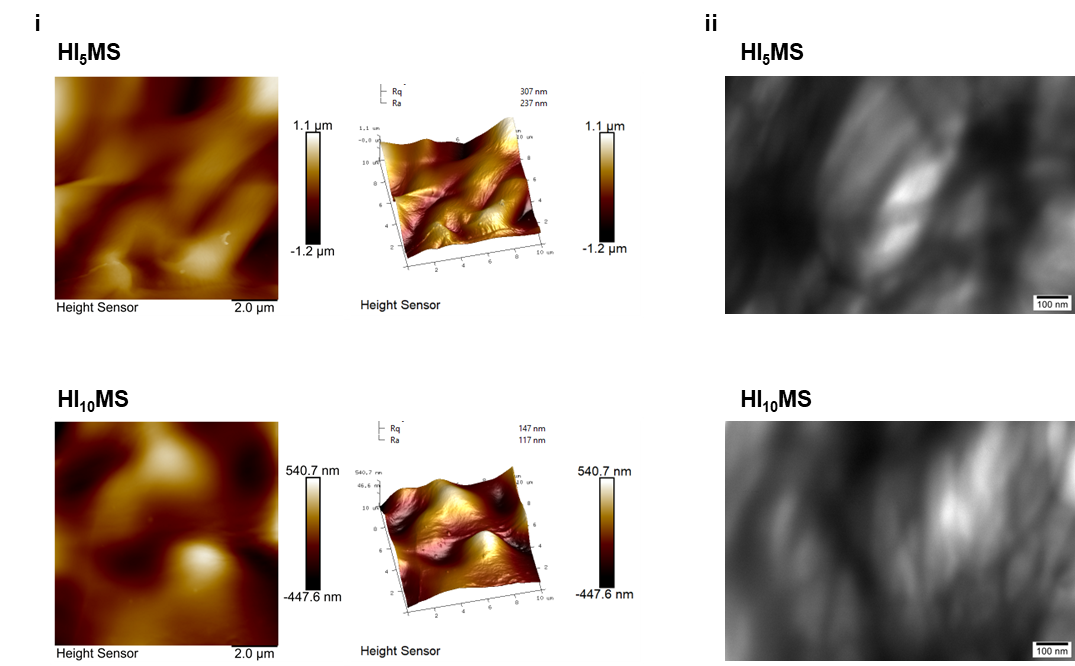
**

**Figure S6. Fluorescence images of rhodamine-labeled NSC-Exos@HIMS.**

**
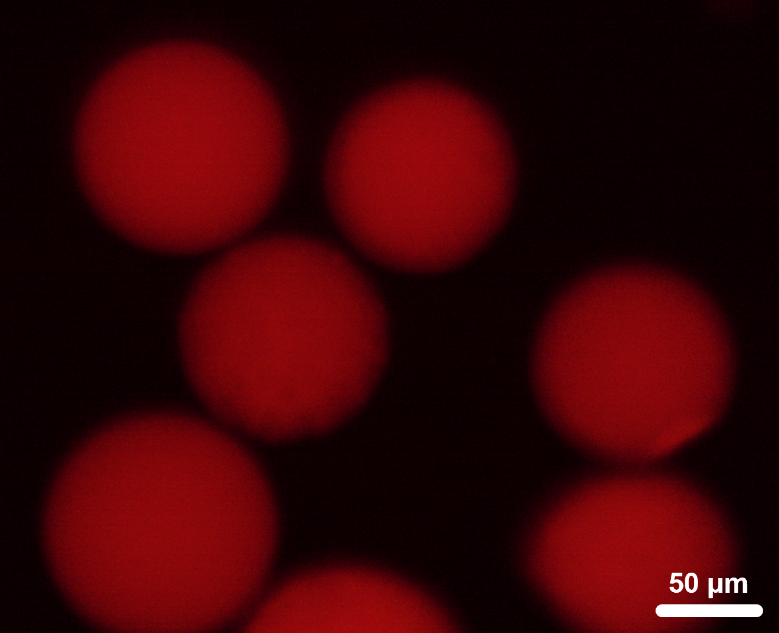
**

**Figure S7. Protein standard curve used to measure exosome concentrations.**

**
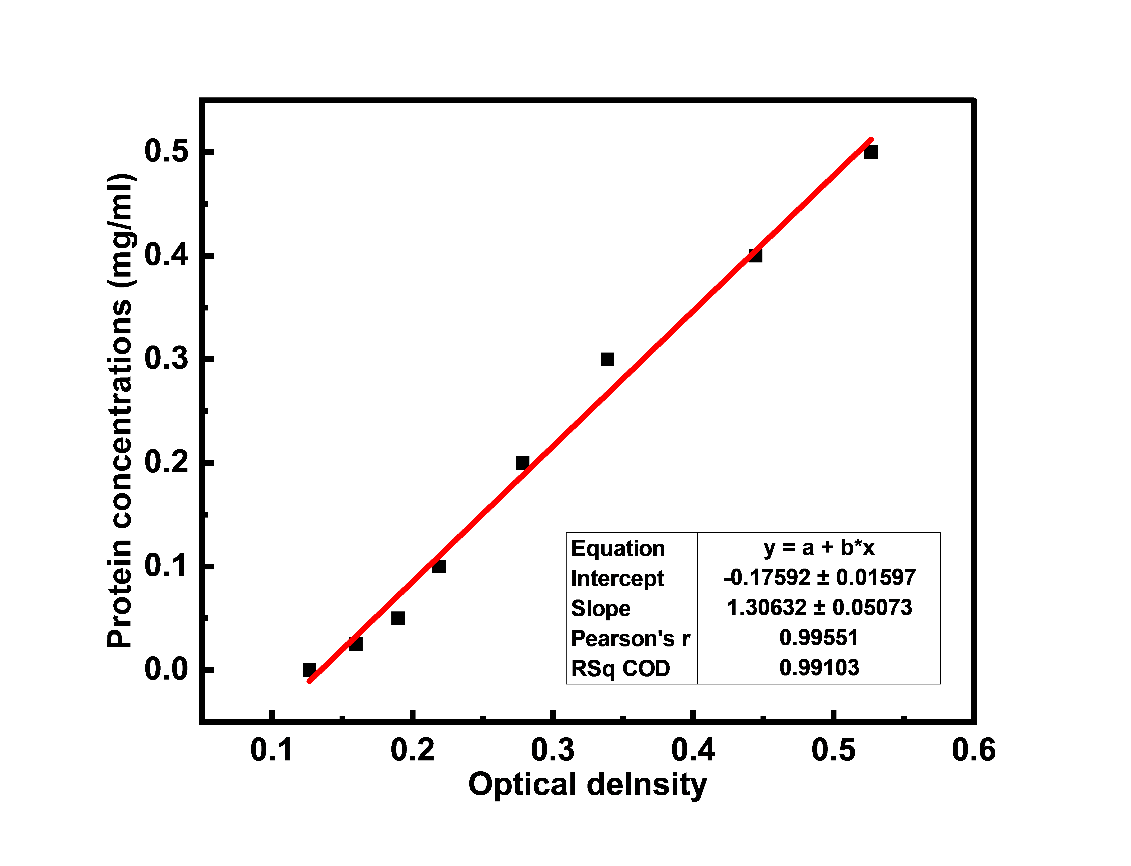
**

**Figure S8. Evaluation of the Biocompatibility of microspheres.** i) Live-dead fluorescence images of cells on different microspheres. ii) Survival rate of cells on different microspheres. (*n* = 3) The data are presented as the mean ± SD. *p < 0.05, **p < 0.01, ****p < 0.001 and ****p < 0.0001.


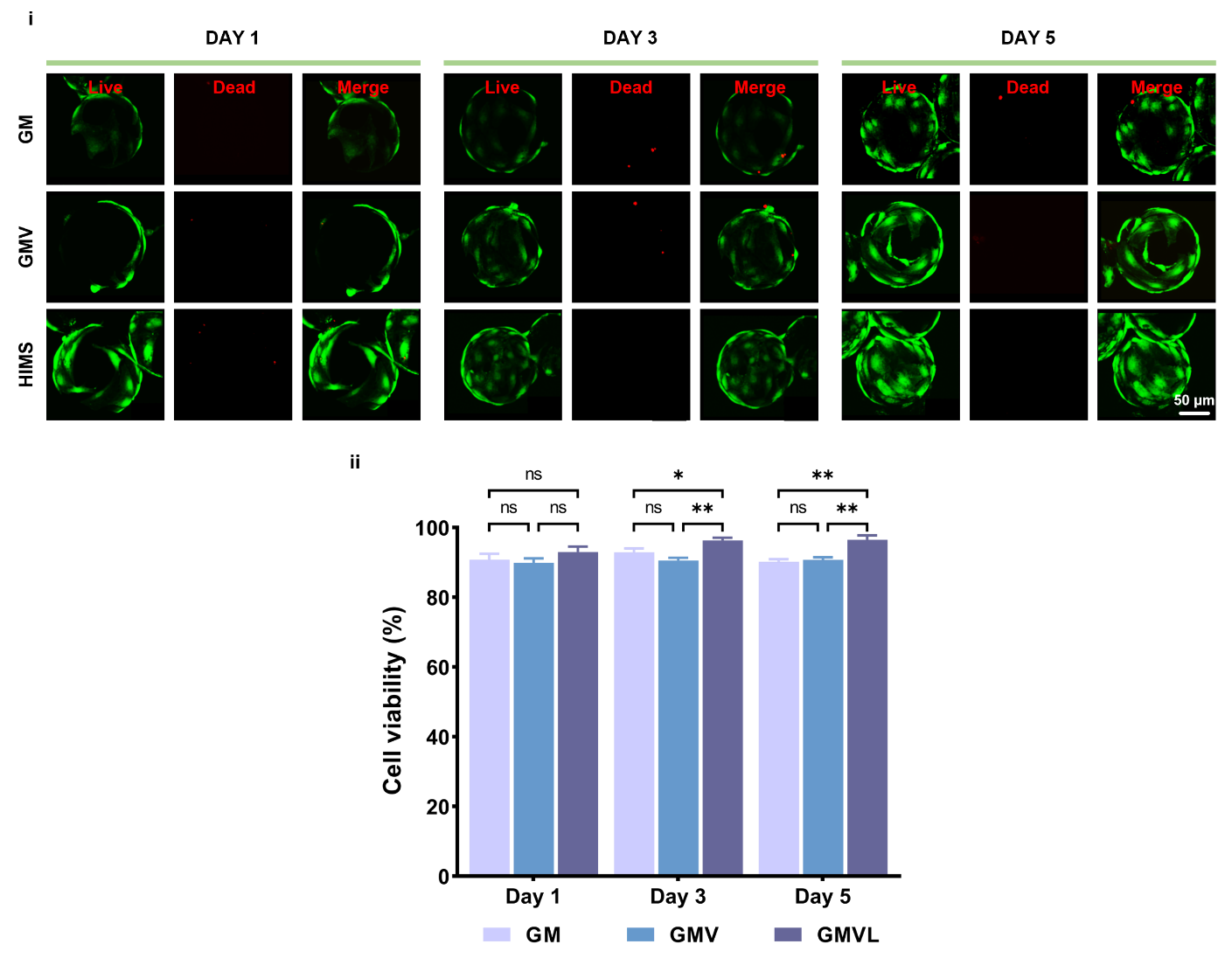


**Figure S9. Western Blot analysis and quantification of HIF-1α protein levels in NPSCs from controls and microspheres.** (*n* = 3) The data are presented as the mean ± SD. *p < 0.05, **p < 0.01, ****p < 0.001 and ****p < 0.0001.

**
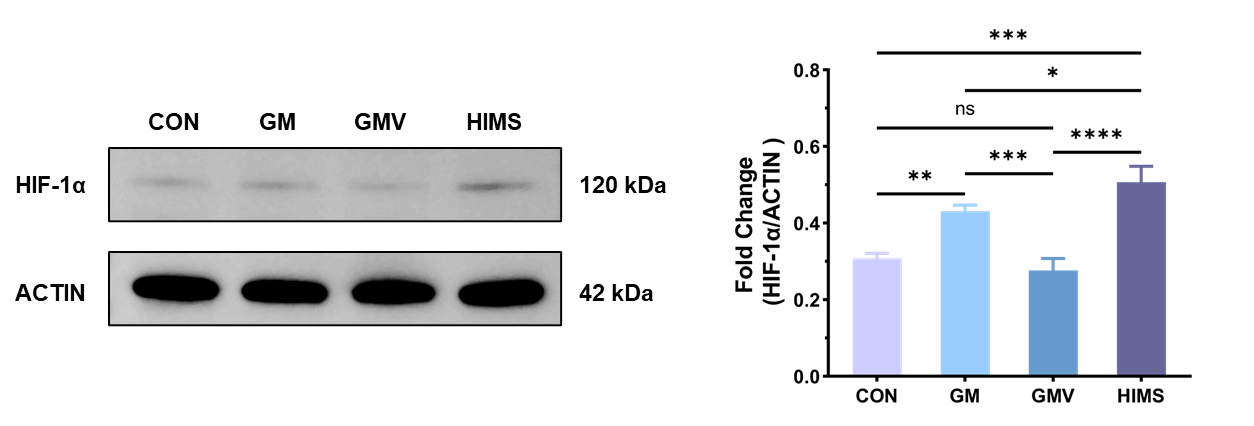
**

**Figure S10. Western Blot analysis and quantification of PI3K (i) and AKT (ii) protein levels in control and hypoxia-induced NPSC.** (*n* = 3) The data are presented as the mean ± SD. *p < 0.05, **p < 0.01, ****p < 0.001 and ****p < 0.0001.


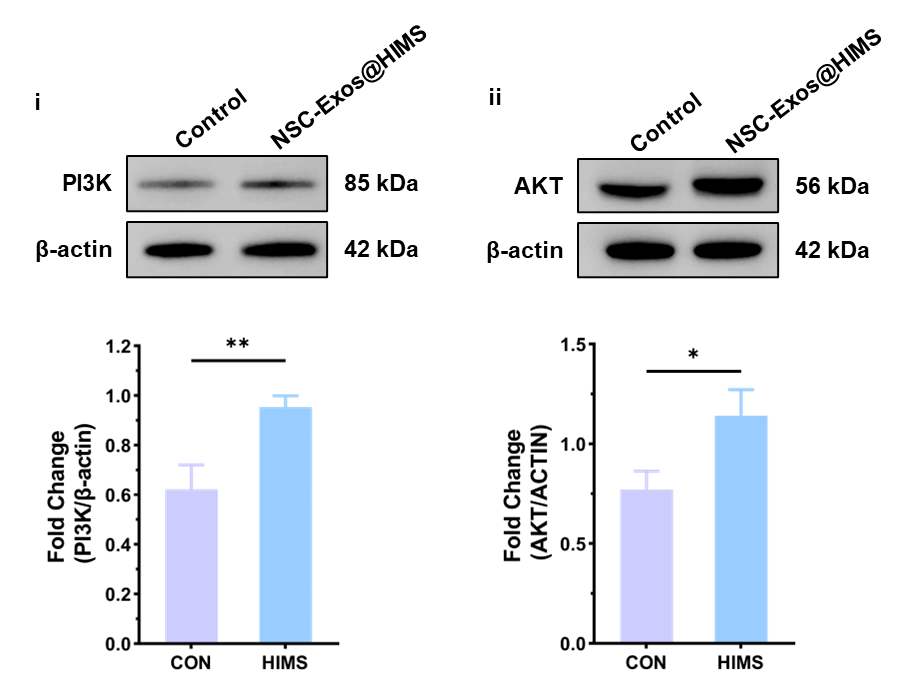

Supplement: Supplementary file 1 — Supporting Information [file ADVS-12-2417570-s001.docx]
